# Ethical and Governance Challenges of AI in Medical Imaging and Diagnostics: A Systematic Survey and Policy Framework Recommendations

**DOI:** 10.3390/healthcare14131975

**Published:** 2026-07-02

**Authors:** Dulani Athukorala, Khandakar Ahmed, Raza Nowrozy

**Affiliations:** College of Engineering and Science, Victoria University, Melbourne 3000, Australia; khandakar.ahmed@vu.edu.au (K.A.); raza.nowrozy@vu.edu.au (R.N.)

**Keywords:** artificial intelligence, medical imaging, diagnostic imaging, ethical AI, AI governance, algorithmic bias, explainability, healthcare regulation, digital health, clinical decision support

## Abstract

**Background/Objectives:** Artificial intelligence (AI) is increasingly embedded within diagnostic imaging workflows, reshaping clinical decision-making, health system governance, and regulatory oversight. While technical advances in radiological AI have accelerated, governance mechanisms have struggled to keep pace with issues of bias, transparency, accountability, and lifecycle oversight. This study examines ethical, regulatory, and implementation challenges in AI-enabled diagnostic imaging, building on prior reviews that have often emphasised technical performance by integrating ethical risk domains with governance responses across the AI lifecycle. **Methods:** This study presents a PRISMA-ScR-informed systematic survey of 156 sources, including peer-reviewed publications, regulatory documents, policy reports, and professional guidance materials (2018–2025), synthesised through thematic analysis and lifecycle mapping spanning data acquisition, model development, deployment, monitoring, and continuous learning. **Results:** Drawing on both thematic insights derived from the reviewed literature and established ethical and regulatory frameworks, we propose a literature-derived conceptual ethical-governance framework organised around five pillars: equity and bias mitigation, explainability and transparency, accountability and oversight, privacy-preserving infrastructure, and adaptive regulatory alignment. Although illustrated through the Australian healthcare context, the framework is designed to be transferable to federated and multi-jurisdictional health systems. This review further identifies trust quantification as an underdeveloped but essential dimension of clinical AI governance, emphasising the need to integrate measurable indicators such as calibration, clinician–AI concordance, and patient acceptance into lifecycle-based evaluation. **Conclusions:** By bridging technical, ethical, and policy perspectives, this review proposes a structured conceptual governance framework to support safe, equitable, and trustworthy AI integration in digital health systems.

## 1. Introduction

In this review, “ethical AI” in diagnostic imaging refers to AI systems that ensure clinical safety and robustness across diverse populations, meaningful transparency for clinician interpretation, documented accountability throughout the AI lifecycle, privacy-preserving data governance, and ongoing monitoring to detect bias, model drift, and performance degradation. This definition operationalises ethical principles into practical governance requirements for the development, deployment, and oversight of clinical AI systems. In recent years, artificial intelligence (AI), which encompasses machine learning (ML), deep learning (DL), and foundation models, has triggered transformative changes in diagnostic healthcare practices [1,2,3,4,5,6,7,8,9,10]. These cutting-edge technologies excel in uncovering diagnostically relevant patterns from high-dimensional medical images, offering levels of accuracy and processing speed that go far beyond traditional approaches. State-of-the-art AI architecture, including convolutional neural networks (CNNs), vision transformers (ViTs), generative adversarial networks (GANs) and large language models (LLMs), have been successfully integrated into clinical imaging workflows, contributing to diverse applications such as anomaly detection, segmentation of anatomical structures, disease classification, radiomic-based feature extraction, individualised risk stratification, and the automation of diagnostic report generation [11,12,13,14,15,16,17,18,19,20,21,22,23,24,25].

Beyond isolated algorithmic performance improvements, AI-enabled diagnostic imaging systems now function as components of broader digital health infrastructures. These systems interact with electronic health records, clinical decision-support platforms, telehealth networks, and national data repositories. Consequently, governance challenges extend beyond model accuracy to encompass interoperability, data governance, institutional accountability, and public trust within digitally connected health ecosystems. Framing AI in diagnostic imaging as a digital health infrastructure component clarifies why lifecycle governance and system-level oversight are essential.

Crucially, trust in clinical AI systems should not be assumed solely on the basis of technical performance or regulatory approval. Instead, trust must be treated as a measurable and context-dependent outcome, requiring evaluation through empirical indicators such as calibration reliability, clinician–AI concordance, and patient acceptance in real-world settings.

### 1.1. Scope and Focus of the Systematic Survey

Although the term *medical imaging* encompasses a broad range of diagnostic modalities, including radiology, histopathology, cytology, and molecular imaging, the primary focus of this systematic survey is on AI-enabled radiological imaging workflows. This emphasis reflects the current maturity of AI deployment, regulatory oversight, and clinical integration within radiology, where AI systems are most widely evaluated, validated, and deployed in practice.

While emerging applications in digital pathology and cytology raise important ethical and governance questions, these modalities are considered outside the core analytical scope of this review. Where relevant, transferable governance implications for non-radiological imaging domains are noted to support broader applicability without overstating coverage. Many of the governance principles identified in this review, including bias mitigation, transparency, accountability, privacy protection, and lifecycle monitoring, are equally relevant to AI applications in pathology and cytology, although their implementation may require adaptation to modality-specific workflows, data characteristics, and diagnostic practices.

AI-driven imaging solutions increasingly demonstrate diagnostic performance comparable to expert clinicians in selected tasks and controlled evaluation settings [1,16,21,26,27,28,29,30]. Multimodal AI systems that integrate imaging data with electronic health records (EHRs), genomics, and clinical narratives signal a paradigm shift toward comprehensive AI-enabled diagnostic ecosystems. However, this technological progress introduces new sociotechnical complexities in relation to transparency, fairness, liability, and governance [27,31,32,33,34,35,36,37,38,39,40,41,42,43,44,45,46,47,48,49]. Opacity remains a central concern; despite high predictive performance, many models operate as “black boxes,” offering limited insight into decision pathways [14,50,51,52,53,54,55,56,57,58,59,60,61,62,63,64,65,66,67,68]. This lack of transparency undermines clinician trust, complicates validation, and introduces medico-legal ambiguity [13,24,31,34,69,70,71,72,73,74,75].

Algorithmic bias further complicates the implementation, as unbalanced training data often perpetuate disparities between demographic groups [76,77,78,79,80,81,82,83,84,85,86,87,88,89,90,91,92,93,94,95,96]. These biases can lead to reduced diagnostic accuracy in minority populations, posing serious ethical and public trust challenges [19,23,44,48,69,70,97,98,99,100]. Thus, systematic governance frameworks for fairness auditing and subgroup validation are urgently needed [101,102,103,104,105,106]. Meanwhile, regulatory responses remain fragmented across jurisdictions. Despite promising advances such as the EU AI Act 2024 and the FDA’s SaMD framework, governance models often lag behind technical developments, especially adaptive, generative or multimodal AI systems [27,32,45,48,70,79,101,103,104,107]. Australia currently combines existing medical device regulation, professional guidance, and voluntary ethical principles; however, important governance challenges remain regarding adaptive AI systems, post-deployment oversight, fairness assurance, and accountability mechanisms [13,46,70,77,100,108,109,110,111,112,113,114,115,116,117]. Existing reviews have frequently focused on technical performance, algorithm development, or isolated ethical and regulatory challenges within diagnostic AI. Comparatively fewer studies have examined how ethical, governance, regulatory, and implementation considerations interact across the full AI lifecycle, from data acquisition and model development to deployment, monitoring, and post-market oversight. Furthermore, the rapid emergence of foundation models, generative AI systems, adaptive algorithms, and evolving regulatory frameworks has created new governance challenges that are not comprehensively captured in earlier reviews. This review addresses these gaps through an integrated synthesis of ethical, governance, regulatory, implementation, and trust-related considerations relevant to contemporary AI-enabled diagnostic imaging systems. To our knowledge, no previous review has comprehensively examined these interconnected dimensions across the full AI lifecycle while simultaneously considering foundation models, generative AI, multimodal systems, and emerging regulatory developments. In response, this systematic survey offers a comprehensive interdisciplinary synthesis of 156 sources (2018–2025), including peer-reviewed publications, regulatory documents, policy reports, and professional guidance materials, across four thematic domains: ethical considerations (52 sources), broader healthcare applications (57 sources), diagnostic imaging implementation (43 sources), and governance, policy, and education (4 sources) [12,71,96,118,119,120,121,122,123,124,125]. This categorisation is summarised in Table 1. The primary evidence corpus comprised sources published between 2018 and 2025. Earlier publications cited throughout the manuscript were used only to provide foundational, historical, conceptual, or regulatory context and were not included in the formal evidence synthesis.

To examine the evolution of research in these domains, Table 2 presents the distribution of articles by year and thematic area from 2018 to 2025. This analysis reveals a steady increase in the level of scholarly attention to the ethical, governance, and policy dimensions of AI integration in medical diagnostics, in addition to continuing advances in broader healthcare applications and diagnostic imaging. These trends highlight the growing recognition that governance frameworks must address not only technical performance but also transparency, fairness, and accountability to ensure responsible and equitable deployment of AI in clinical practice.

### 1.2. Comparison with Previous Studies (2018–2025)

Although several reviews between 2018 and 2025 have examined AI in medical imaging, most have focused narrowly on technical performance, specific ethical concerns, or isolated governance aspects [16,23,24,103]. Few provide a comprehensive synthesis that integrates these dimensions between different modalities and jurisdictions [1,13,21]. This systematic survey addresses this gap by analysing 156 sources published between 2018 and 2025 and categorising them into ethical considerations, healthcare applications, diagnostic implementation, and governance frameworks. The trends captured in Table 2 reveal a growing shift toward ethical and policy-oriented concerns along with technical advances. Crucially, previous studies often overlooked emerging AI paradigms, such as foundation models, generative AI, multimodal fusion, and federated learning, which introduce new ethical and regulatory complexities [72,121,122]. Our analysis incorporates these developments, assessing their impact on key ethical and governance concerns. Although earlier reviews typically stop at general ethical discussions [31,32,70], we advance the field by offering concrete policy recommendations tailored to the Australian healthcare system, emphasising regulatory standardisation, adaptive oversight, fairness auditing, and stakeholder accountability.

### 1.3. The Role of AI in Medical Imaging

The integration of artificial intelligence (AI) into medical imaging represents one of the most consequential evolutions in diagnostic medicine [15,26,69,126,127,128,129,130,131,132]. Using large-scale annotated datasets and advanced computational architecture—such as convolutional neural networks (CNN), recurrent models, vision transformers, and generative frameworks—AI systems are capable of learning abstract, high-dimensional feature representations directly from raw pixel data [1,6,13,21,54]. These representations allow automated systems to perform complex image-based tasks with high fidelity, ranging from anatomical segmentation and lesion detection to disease classification, risk stratification, and outcome prediction [26,70,106,109,110,111,112,113,114,115,116,117,119,133,134].

#### 1.3.1. Automated Disease Detection and Diagnosis

AI applications in medical imaging have achieved benchmark performance in numerous specialties. In oncology, AI models can detect pulmonary nodules, breast lesions, and brain tumours with high sensitivity, often outperforming average radiologists in retrospective settings [1,12,13,15,16]. Similar success has been reported in cardiology and neurology, where deep learning systems identify ischaemic strokes, atherosclerotic plaque, and aneurysms from CT angiography and MRI scans [11,21,54,132,135]. In the context of infectious diseases, such as COVID-19, artificial intelligence has been deployed for the rapid detection of viral pneumonia from chest radiographs and lung CT, demonstrating clinical utility in the triage during high volumes of patients [11,13,98,100,107,108,135,136,137,138].

#### 1.3.2. Radiomics and Prognostic Modelling

Beyond detection, AI enables radiomics, a domain focused on extracting and analysing quantitative imaging features that are imperceptible to the human eye [1,15,16,73,135]. These features, when combined with patient-specific clinical data, support precision diagnostics by identifying phenotypic signatures associated with treatment response, tumour progression, or survival outcomes. Predictive models trained on radiometric inputs are increasingly used in stratifying cancer patients, guiding the selection of personalised therapy, and forecasting the risk of recurrence in colorectal, breast, and prostate cancers [11,13,18,27,61,98,100,107,108,133,134,135,136,137,138].

#### 1.3.3. Workflow Optimisation and Operational Efficiency

AI systems also contribute to efficiency in clinical workflows. Applications include automated case triage based on acuity, prioritisation of emergent conditions (e.g., intracranial haemorrhage), real-time quality control in imaging acquisition, and the generation of structured reports using natural language processing (NLP) [27,36,42,59,139]. AI-powered integration with Picture Archiving and Communication Systems (PACS) and Radiology Information Systems (RIS) can reduce reporting delays, improve workflow efficiency, and mitigate human error. These systems increasingly support multimodal imaging analysis and the fusion of images with clinical notes or omics data, enabling more holistic diagnostic insights [9,12,13,72,127].

#### 1.3.4. Emerging Frontiers: Multimodal, Federated, and Generative Models

The next generation of AI imaging applications involves multimodal fusion: combining radiological data with genomics, pathology slides, or electronic health records to build richer diagnostic models [27,67,69,72,140]. Moreover, federated learning allows AI models to train in decentralised hospital datasets without compromising patient privacy, aligning with global data protection laws such as GDPR [25,27,44,120,141]. Generative adversarial networks (GANs) are also being explored to create synthetic training data sets, expand minority cases, and address bias by balancing underrepresented pathologies [72,98,121,122,125].

### 1.4. Ethical and Regulatory Challenges

The integration of artificial intelligence (AI) into medical imaging amplifies long-standing ethical concerns while introducing new regulatory complexities beyond traditional healthcare governance [11,12,26,70,79,101,102,103,104,105,106,142]. Across the reviewed literature, ethical concerns consistently clustered around five recurring domains: subgroup bias and inequitable performance, limited model interpretability in high-stakes clinical contexts, ambiguous accountability between clinicians and AI systems, privacy vulnerabilities in multi-institutional data ecosystems, and regulatory fragmentation across jurisdictions. While these concerns are widely acknowledged, operational mechanisms for systematic enforcement and lifecycle oversight remain comparatively underdeveloped [25,32,46,48,70,77,108,130,143].

As shown in Table 3, this taxonomy demonstrates that while technical solutions addressing bias, explainability, and privacy are frequently proposed, their validation often remains retrospective rather than prospective. In contrast, governance mechanisms for post-deployment monitoring and adaptive model updates are comparatively underdeveloped. The distribution of validation maturity across lifecycle stages reveals a structural imbalance: innovation in algorithmic capability frequently outpaces institutional readiness for oversight and accountability.

Table 3 demonstrates that while numerous technical and governance interventions have been proposed across the AI lifecycle, many remain supported primarily by retrospective, pilot, simulation, or policy-level evidence, highlighting the need for stronger prospective clinical validation and post-deployment evaluation.

#### 1.4.1. Algorithmic Bias and Fairness

Bias often arises from unrepresentative data, skewed sampling, and feedback loops that reinforce disparities, disproportionately affecting marginalised groups [6,23,69,98,100,106,125,137]. Fairness requires inclusive datasets, continuous bias audits, demographic performance reporting, and active stakeholder engagement throughout development [19,24,26,74,143,144].

#### 1.4.2. Transparency and Explainability

Deep learning models often lack interpretability, compromising clinician trust, informed consent, and accountability [34,50,73,117,124,126,127]. Clinicians need not only accurate predictions but clear reasoning, especially in critical cases such as oncology and surgical planning [24,40,72,77,144]. Tools such as saliency maps, LIME, SHAP, and counterfactuals show promise but remain inconsistent and lack clinical validation standards [14,25,72,73,119,126,134].

#### 1.4.3. Accountability and Liability

Current legal frameworks struggle to assign responsibility when AI errors cause harm [31,33,79,100,101,145]. This is particularly urgent for autonomous or semi-autonomous systems, such as triage algorithms [12,51,103,114]. Clear accountability frameworks are needed, including audit trails, consent processes that recognise algorithmic uncertainty, and third-party validation in clinical settings [17,32,107,108].

#### 1.4.4. Regulatory Fragmentation and Global Inconsistencies

Disparities between regulatory bodies such as the FDA, EMA, and Australia’s TGA complicate the oversight of AI-enabled medical devices [45,70,103,104,107,108]. Jurisdictions differ particularly in the regulation of adaptive models that evolve after deployment [35,46,48,70,146]. Meanwhile, ethics frameworks often do not keep pace with technology, weakening transparency and accountability [23,49,50,73,86,100].

**Figure 1:**
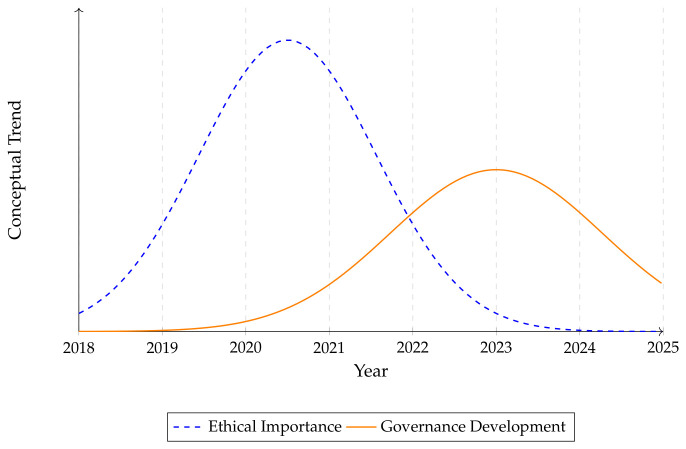
Conceptual illustration of the evolving relationship between ethical concerns and governance development in AI-enabled medical imaging between 2018 and 2025, based on thematic patterns identified in the reviewed literature.

These challenges highlight the need for globally coordinated and interdisciplinary governance. Equitable data governance, participatory policy design, and ethically embedded technical development are crucial [71,96,118,119,120,121,122,123,124,125]. As conceptually illustrated in Figure 1, the reviewed literature suggests that ethical concerns surrounding AI in medical imaging became prominent earlier than the development of formal governance frameworks, highlighting a persistent gap between ethical expectations and regulatory readiness.

### 1.5. Comparative Analysis of Global Governance Frameworks

The governance of AI in healthcare is evolving unevenly across jurisdictions, shaped by regional policy priorities, institutional capacity, and historical regulatory paradigms. Although some regions are adopting proactive, risk-based legislation (e.g., the European Union AI Act 2024), others continue to operate through legacy medical device pathways or advisory ethical frameworks. This regulatory fragmentation creates a significant asymmetry in legal enforceability, ethical accountability, and innovation scalability [32,70,101,104,107].

In all jurisdictions, common goals have emerged such as human oversight, explainability, transparency, and patient safety. However, the degree to which these goals are legally binding, operationalised, and aligned with clinical realities remains inconsistent. Emerging issues such as adaptive algorithmic updates, synthetic data training, and cross-border compliance raise additional complexity for regulators worldwide [6,27,36,45,77,127]. A comparative overview of AI governance strategies related to healthcare from ten representative frameworks is presented in a later section of this review. This comparison incorporates both binding legislation and normative ethical guidelines. This comparative lens underscores the need for transnational coordination, agile co-regulation models, and standards for continuous algorithmic validation—particularly as AI systems transition from static tools to dynamic, learning entities deployed at scale [15,26,69,79,100,101,107,108,126,127,128,129,130,131,132].

Artificial intelligence (AI) is rapidly transforming medical imaging by enhancing diagnostic precision, reducing clinician burden, and optimising radiological workflows [11,12]. During the past decade, AI techniques, particularly deep learning (DL), convolutional neural networks (CNNs), and natural language processing (NLP), have enabled automated image classification, lesion detection, radiomics, and clinical report generation [70]. Although these capabilities have achieved state-of-the-art performance in various tasks, they also raise urgent ethical and regulatory concerns [13,34,69,101,142]. In the context of the integration of AI into radiology and diagnostic decision making, it is critical to establish governance mechanisms that address societal concerns and clinical safety. Current debates around artificial intelligence in healthcare protocols increasingly emphasise not only technical precision but also the development of systems that are explicable, equitable, and ethically grounded [11,46,97].

### 1.6. Key Advances in AI-Driven Medical Imaging

The application of AI in medical imaging has evolved from discrete detection tasks to complete, end-to-end diagnostic support systems. These systems combine computer vision techniques with clinical reasoning tools, underpinned by convolutional neural networks (CNNs), transformers, radiomics, and large language models (LLMs) [11,12,13,15,16,71,90].

- **Automated Image Interpretation:** AI models now reliably identify tumours, fractures, nodules, and haemorrhages from CT, MRI, PET and X-ray scans, performing on the same level as experienced radiologists in many settings [1,40,105].
- **Radiomics and Predictive Modelling:** Advanced radiomic workflows extract latent image features to stratify patient risk, predict recurrence, and assess therapeutic responsiveness in oncology and neurology [16,70,73].
- **Clinical Workflow Optimisation:** AI augments PACS/RIS systems by prioritising emerging findings, automating measurements, and populating structured radiology reports, thus improving operational efficiency [27,36,139].
- **Multimodal Integration via Foundation Models:** Emerging LLM-powered platforms synthesise textual and imaging data, allowing queries in natural language, diagnostic summarisation, and report generation [71,90,140].

AI models serve as intermediaries between raw image data and downstream diagnostic decision making. Feedback loops ensure continuous learning and calibration of model predictions based on the input of the radiologist, enhancing robustness and reliability [15,25,40,127].

### 1.7. Visualising AI’s Role in Medical Imaging

**Figure 2:**
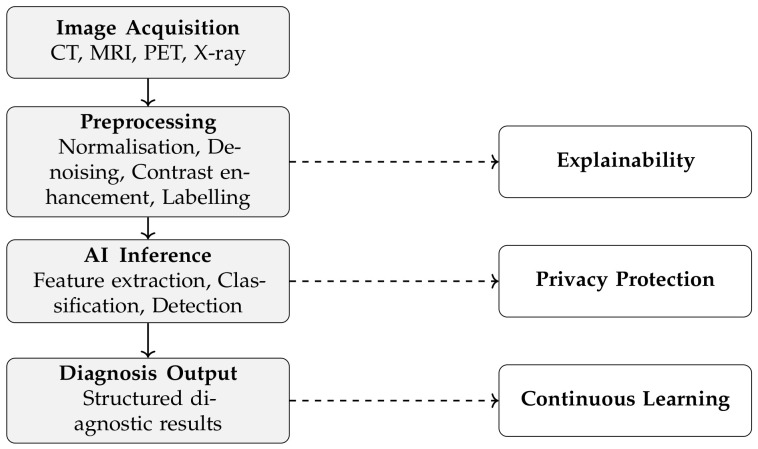
Modular AI pipeline for medical imaging illustrating the progression from image acquisition to diagnostic output and highlighting key governance considerations, including explainability, privacy protection, and continuous learning.

To capture the intricacies of AI integration within diagnostic imaging, Figure 2 presents a comprehensive modular pipeline that represents the flow from raw image acquisition to clinical decision support. This enhanced schematic emphasises not only the technical progression of the data, but also the integration of essential components such as explainability, privacy, and continuous learning, reflecting current research trends and regulatory priorities. The workflow begins with *Image Acquisition*, where imaging modalities such as CT, MRI, PET, or X-ray produce diagnostic scans. These raw images are then passed to the *Preprocessing* layer, which applies normalisation, denoising, and contrast enhancement to ensure input standardisation and reduce artefacts. This step may also include anatomical labelling and harmonisation, especially in multicentre deployments [71,74,96,97,99,118,119,120,121,122,123,124,125,139,143,144,146,147,148,149].

Following preprocessing, data enter the AI Inference Engine, which may consist of convolutional neural networks (CNNs), transformers, or large multimodal language models (LLMs). These models perform critical computational tasks such as tumour segmentation, anomaly detection, risk stratification, and report generation. In parallel, an integrated explainability module operates, employing techniques such as SHAP, LIME, saliency maps, and counterfactuals to generate human-understandable justifications for model outputs. This is essential for transparency, regulatory compliance, and the promotion of patient trust [27,41,42,43,44,45,46,47,48,49]. A dedicated Privacy Protection Layer protects sensitive health data through methods such as federated learning, differential privacy, and homomorphic encryption, ensuring compliance with GDPR, HIPAA, and related standards without degrading AI performance. The output of the inference engine is then delivered to a Diagnosis Output unit, which produces structured results (e.g., classifications, segmentations, heatmaps) for clinical interpretation. These results are seamlessly integrated into hospital systems such as PACS (Picture Archiving and Communication Systems) or RIS (Radiology Information Systems) through a Clinical Decision Support module, allowing real-time triage, prioritisation of urgent findings, and partially or fully automated reporting [70,109,110,111,112,113,114,115,116,117].

A crucial component of this pipeline is the human-in-the-loop feedback system, in which radiologists review AI-generated outputs, validate results, and provide corrective feedback. This continuous feedback loop supports model retraining, mitigates performance drift, and promotes ongoing learning aligned with evolving clinical practice. By incorporating explainability, privacy safeguards, and feedback-driven iteration, the system examines not only technical robustness, but also ethical, legal, and social requirements essential for reliable AI deployment in healthcare. Figure 2 shows a generalised workflow that illustrates the full pipeline: from raw medical image acquisition (MRI, CT, X-ray) through preprocessing and feature extraction to AI-driven analysis and diagnostic output. The modular structure reflects the interdependent stages of AI-assisted diagnostic radiology, underscoring the need for data integrity, transparency, and accountability at each step [76,77,78,79,80,81,82,83,84,85]. Table 4 summarises this functional architecture, detailing how data flow across acquisition, preprocessing, inference, and decision support while embedding safeguards for explainability, privacy, and continuous learning. The deployment of AI in medical imaging, while technically transformative, demands a robust and multilayered governance framework to ensure ethical sustainability and clinical safety. Governance must extend beyond voluntary codes or retrospective audits, establishing enforceable mechanisms guiding the entire AI lifecycle, from data acquisition and model development to deployment, ongoing monitoring, and decommissioning. This framework must remain proactive and adaptive, ready to address the emerging challenges of rapidly evolving technologies such as LLM and generative AI [72,73,86,87,88,89,90,91,92,93,94,95,133,134,140,141,150,151,152,153].

Table 4 illustrates that ethical and governance considerations emerge throughout the AI-enabled diagnostic workflow. These considerations extend from data acquisition and preprocessing to model explainability, clinical deployment, and post-deployment learning processes.

As illustrated in Figure 2, governance considerations are not confined to a single stage of the workflow but must be embedded throughout the AI lifecycle, spanning data acquisition, model development, deployment, and post-deployment monitoring.

### 1.8. Derivation of the Conceptual Governance Framework from the Surveyed Literature

The five-pillar conceptual ethical governance framework proposed in this survey is derived from the lifecycle-centric synthesis of ethical risks and governance responses identified across the reviewed literature. As the framework emerged from thematic synthesis of the included evidence rather than empirical implementation or prospective clinical validation, it is presented as a conceptual governance framework intended to organise and operationalise governance requirements identified across the evidence base. Drawing on the taxonomy presented in Table 5, recurring governance gaps were traced across data curation, model development, validation, clinical deployment, and post-deployment monitoring stages. The resulting pillars reflect intervention points consistently emphasised across studies, rather than abstract normative principles introduced independently of the survey findings.

The proposed conceptual framework does not seek to introduce entirely new ethical principles. Rather, it synthesises and operationalises governance requirements repeatedly identified across the reviewed literature and aligns them with established international guidance. Existing frameworks, including the WHO Guidance on Ethics and Governance of Artificial Intelligence for Health, OECD AI Principles, FDA Good Machine Learning Practice guidance, the European Union AI Act, and Australian TGA recommendations, provide important ethical principles, regulatory obligations, and risk-management expectations. However, these frameworks are predominantly presented as principle-based, regulatory, or risk-classification approaches and provide limited guidance on how governance activities should be systematically integrated across the full AI lifecycle.

The principal contribution of the proposed conceptual framework lies in its lifecycle-centric organisation of governance requirements. By mapping ethical risks, governance responses, accountability mechanisms, and trust indicators to specific stages of the AI lifecycle, the framework translates broad ethical principles into operational governance activities. This structure enables continuous oversight across data acquisition, model development, validation, clinical deployment, post-deployment monitoring, and adaptive system updating. In doing so, the framework provides a practical mechanism for identifying governance gaps, assigning accountability, and supporting trust calibration throughout the lifecycle of AI-enabled diagnostic systems.

Table 5 demonstrates that ethical risks, governance requirements, and trust indicators vary across the AI lifecycle, reinforcing the need for stage-specific oversight mechanisms rather than a single governance approach applied uniformly across all phases of AI development and deployment.

Table 6 maps each governance pillar to the evidence signals identified across the reviewed literature (n = 156), their relative prevalence, and the principal implementation gaps reported in the evidence base.

1. **Bias Mitigation and Equity Auditing**
  - Address algorithmic disparities caused by skewed or incomplete training data.
  - Mitigate diagnostic bias between race, gender, and age groups [69,99,100].
  - Insist on equity audits, demographic benchmarking, and subgroup performance reporting.
2. **Explainability and Transparency**
  - Combat the opacity of black-box models (e.g., deep neural networks).
  - Integrate explainable AI (XAI) tools saliency maps, SHAP values, counterfactuals to clarify predictions [26,34,73].
  - Make interpretability accessible to both clinicians and patients.
3. **Interdisciplinary and Participatory Oversight**
  - Involve a broad stakeholder base: Clinicians, ethicists, patients, legal experts, and developers.
  - Promote ethical co-design to improve foresight and system resilience [46,86,97].
4. **Privacy-Preserving Data Infrastructure**
  - Embed privacy by design using secure techniques: federated learning, differential privacy, and homomorphic encryption [27,44,120].
  - Ensure compliance with data protection laws (e.g., GDPR, HIPAA) and minimise re-identification risk.
5. **Regulatory and Policy Alignment**
  - Harmonise ethical AI practices with global frameworks: EU AI Act 2024, FDA SaMD guidelines, WHO recommendations [70,101,107].
  - In Australia, bridge policy gaps by advancing national regulatory standards.

The prevalence classifications reflect thematic frequency across the 156 reviewed studies. Ethical concerns related to bias and fairness were widely discussed, whereas structured governance mechanisms for post-deployment monitoring and adaptive systems were comparatively underdeveloped. This distribution highlights a recurring pattern in the literature: normative recognition of risks often exceeds operationalised governance implementation.

### 1.9. Real-World Deployment and Governance Challenges in Clinical Practice

While much of the surveyed literature focuses on anticipated ethical risks, several real-world deployment experiences illustrate how governance gaps materialise in clinical settings. These cases highlight the importance of lifecycle-aware oversight and reinforce the practical relevance of the proposed governance framework.

#### 1.9.1. Case 1: Performance Degradation and Post-Market Oversight

Several AI-enabled radiology tools have demonstrated reduced diagnostic performance when deployed outside their original training environments, particularly when applied across different patient populations or imaging devices. Such cases expose governance gaps at the post-deployment monitoring stage, where model drift and context shift are insufficiently addressed by static approval mechanisms. These experiences underscore the need for continuous performance auditing and adaptive oversight, directly motivating the framework’s emphasis on lifecycle-aware governance and accountability structures.

#### 1.9.2. Case 2: Automation Bias and Clinical Responsibility

Reports of clinicians over-relying on AI-generated outputs in diagnostic workflows have raised concerns regarding automation bias and unclear responsibility boundaries. In these instances, AI recommendations were followed despite conflicting clinical judgement, revealing weaknesses in human-in-the-loop governance during the clinical deployment stage. The literature consistently identifies the absence of clear role delineation and accountability protocols as contributing factors, reinforcing the framework’s focus on clinician oversight, responsibility allocation, and institutional governance safeguards.

#### 1.9.3. Case 3: Data Governance and Secondary Use Risks

Real-world controversies surrounding the secondary use of medical imaging data for AI development without meaningful patient awareness or consent have highlighted persistent data governance failures. These cases, often emerging during data curation and post-deployment data reuse stages, demonstrate how privacy risks extend beyond initial model development. Such examples support the framework’s prioritisation of consent-aware data stewardship and transparent governance mechanisms that extend across the full AI lifecycle.

- **Opacity and the Epistemic Black Box:** Clinicians often report difficulty in interpreting model predictions or assessing their reliability in atypical cases [13,34,71,72]. This epistemic opacity undermines informed consent, impedes error investigation, and complicates medico-legal accountability.
- **Algorithmic Fairness and Diagnostic Disparities:** The lack of fairness metrics in performance reporting perpetuates these blind spots. Bias can also arise from equipment heterogeneity, annotation subjectivity, and socioeconomic data gaps [69,97,99].
- **Unclear Legal and Professional Accountability:** Should a misclassification be attributed to the radiologist, the AI developer, or the deploying hospital? Current legal frameworks remain inadequate to address these distributed responsibilities [2,19,31,33]. Without clear attribution mechanisms, accountability becomes diffused, increasing the risk of untraceable harm.
- **Privacy, Consent, and Data Sovereignty:** The use of data in AI training and inference requires stringent privacy safeguards. However, many systems are built on retrospective datasets collected without explicit consent for AI use [27,44,101,120]. Furthermore, cloud-based services used to train or deploy AI models can transfer data across jurisdictions, raising concerns about data sovereignty and legal exposure.
- **Regulatory Ambiguity and Lifecycle Oversight:** Regulatory frameworks have yet to fully address the continuous evolution of AI models, particularly those that update after deployment through online learning or user feedback. This adaptive nature introduces new risks, such as concept drift and unintended behaviour changes that escape initial validation [11,46,70,107,108,142]. The absence of standardised lifecycle governance that covers monitoring, revalidation, and retirement leaves a critical oversight gap.
- **Risk of Clinical Deskilling and Over-Reliance:** Studies have shown that frequent deferral to automated recommendations can diminish diagnostic intuition and reduce the likelihood of independent verification, especially under time constraints [41,42,139]. This phenomenon raises long-term questions about professional competence and patient safety.
- **Socio-ethical Disconnects and Lack of Public Trust:** The socioethical disconnect contributes to mistrust, particularly when patients do not know that their care is being influenced by algorithms [44,50,58]. Without transparent communication and participatory design, AI risks deepening existing fault lines in the patient–clinician relationship.

**Figure 3:**
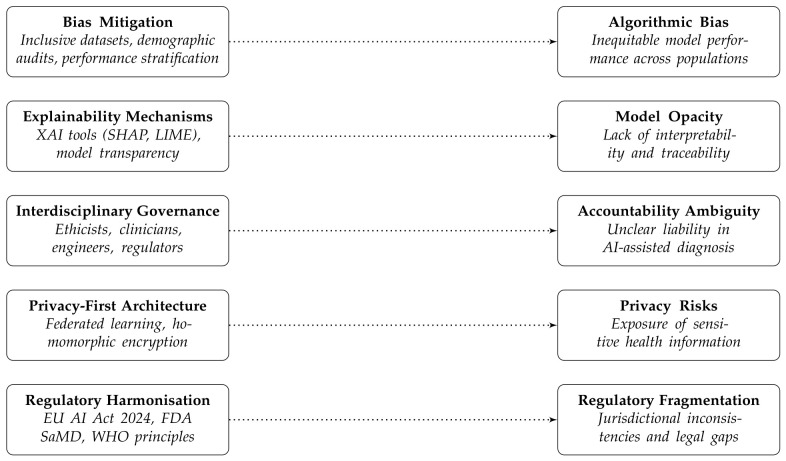
Conceptual alignment between ethical governance mechanisms and the principal ethical and operational challenges encountered in AI-enabled medical imaging.

These ethical challenges are not static; they evolve as AI systems become more sophisticated and embedded in clinical infrastructure. Addressing them requires not only technical interventions, but also institutional readiness, legal reform, and cultural shifts toward transparency and inclusion. Governance, therefore, must operate at the intersection of ethics, policy, and real-world clinical practice. Figure 3 presents a conceptual alignment between the core governance mechanisms and the main ethical challenges associated with AI-enabled medical imaging. It consists of two parallel vertical structures: the left side highlights five foundational pillars of ethical governance, and the right side lists the major ethical challenges commonly encountered in AI deployments. Each directional arrow represents a targeted intervention by which a governance component is intended to mitigate or directly address a corresponding risk or limitation.

Figure 3 illustrates that effective governance requires targeted interventions that directly address specific ethical and operational risks, rather than relying on generic governance controls applied uniformly across the AI lifecycle.

- Bias Mitigation (Algorithmic Bias). Bias mitigation strategies involve curating demographically inclusive datasets and applying subgroup-level performance tests to prevent discriminatory results. This governance pillar responds to algorithmic bias, a challenge stemming from unbalanced or non-representative training data, which may lead to diagnostic inaccuracies in under-represented groups [69,99,121,122,123,124,125].
- Explainability (Opacity of AI Systems). The use of model-agnostic interpretability techniques such as SHAP (Shapley Additive Explanations), LIME (Local Interpretable Model-Agnostic Explanations), and saliency maps aims to make opaque AI systems clearer to clinicians. This evaluates the lack of interpretability associated with black-box models, particularly deep neural networks, and supports informed clinical use and regulatory scrutiny [26,73,97,99,139,144,147,148].
- Interdisciplinary Oversight (Accountability Ambiguity). Establishing shared governance across clinical, ethical, legal and technical domains enables clarity around responsibility and liability in AI-enabled diagnostic pathways. This is critical to solving the accountability vacuum that arises when AI errors occur, especially when models operate autonomously or in critical contexts [31,79].
- Privacy-first infrastructure (data privacy violations). Federated learning, differential privacy, and homomorphic encryption are part of a privacy-first architectural approach that protects sensitive patient data during AI training and deployment. These safeguards directly respond to concerns about unauthorised access and reidentification risks in distributed or cloud-based imaging environments [27,44].
- Policy alignment (Regulatory fragmentation). Policy harmonisation with frameworks such as the EU AI Act 2024, the US FDA’s SaMD Action Plan and WHO’s AI ethics guidance is vital to bridge inconsistencies between jurisdictions in regulation. This ensures consistent lifecycle oversight, improves interoperability, and fosters transnational trust in AI systems deployed in various health systems [70,107]. The figure underscores how a proactive and modular governance strategy can preemptively mitigate downstream risks, creating the ethical infrastructure necessary for a responsible, scalable, and clinically safe deployment of AI in medical imaging.

Taken together, these ethical challenges do not arise in isolation but emerge across the full AI lifecycle in medical imaging, from data acquisition and model development to deployment and post-market monitoring. Issues frequently co-occur, reinforcing one another and complicating clinical governance. Rather than treating these concerns as discrete risks, the literature increasingly frames them as interdependent sociotechnical failures requiring coordinated regulatory, institutional, and technical responses.

## 2. Review Plan and Conduct

This systematic survey of the literature was conducted and reported in accordance with the Preferred Reporting Items for Systematic Reviews and Meta-Analyses Extension for Scoping Reviews (PRISMA-ScR) guidelines where applicable to interdisciplinary governance-focused evidence synthesis.

Although this study employed a systematic search strategy, predefined eligibility criteria, and structured evidence synthesis procedures, PRISMA-ScR reporting guidance was selected instead of the standard PRISMA framework because the primary objective of this review was to map and synthesise a broad and heterogeneous body of evidence rather than evaluate the effectiveness of a specific intervention or undertake quantitative outcome comparison. The review incorporated diverse evidence sources, including peer-reviewed studies, regulatory documents, policy reports, and professional guidance materials spanning clinical, technical, ethical, and governance domains. Given this interdisciplinary scope and the emphasis on identifying, categorising, and synthesising ethical and governance challenges across the AI lifecycle, PRISMA-ScR was considered more appropriate than standard PRISMA, which is primarily designed for systematic reviews focused on intervention effectiveness and quantitative evidence synthesis. Accordingly, this study is presented as a systematic survey of the literature informed by PRISMA-ScR reporting principles.

A completed PRISMA-ScR checklist, including corresponding manuscript page references, has been provided as Supplementary File S1 to support reporting transparency and facilitate assessment of compliance with PRISMA-ScR reporting recommendations. Full database-specific search strategies and corresponding search dates have been provided in Supplementary File S2 to enhance methodological transparency and reproducibility.

While systematic search, screening, eligibility assessment, and structured synthesis procedures were employed, the review was designed to identify, classify, and synthesise heterogeneous evidence relating to ethical, governance, regulatory, technical, and implementation challenges in AI-enabled diagnostic imaging. Consistent with the objectives of a systematic survey, the emphasis was placed on evidence mapping, thematic synthesis, and governance analysis across diverse evidence types rather than quantitative outcome comparison or intervention-effectiveness assessment.

The methodology was designed to support interdisciplinary thematic synthesis and lifecycle-oriented governance analysis across scholarly, clinical, ethical, and regulatory literature relating to AI-enabled diagnostic imaging. This research design was selected because the study sought to systematically identify, classify, and synthesise heterogeneous evidence spanning technical, clinical, ethical, governance, and regulatory domains. A PRISMA-ScR-informed systematic survey was considered the most appropriate approach for addressing the research questions because it enables broad evidence mapping, thematic synthesis, and lifecycle-oriented governance analysis across diverse evidence types that would not be suitable for quantitative review or meta-analysis.

To ensure a robust and thematically comprehensive methodological foundation for this review, this section details the systematic approach used in the identification, selection, classification, and synthesis of the relevant literature. Given the interdisciplinary nature of AI in medical imaging, spanning computer science, clinical medicine, ethics, and regulatory science, the review protocol was designed to accommodate heterogeneity across sources while preserving reproducibility and conceptual coherence.

The review was designed to capture not only technical model development but also scholarship addressing AI integration within digital health systems, including regulatory policy, health informatics governance, clinical workflow redesign, and institutional accountability structures.

### 2.1. Objectives and Research Questions

The overarching objective of this review is to investigate the ethical and governance implications of AI integration in diagnostic medical imaging. The study is guided by the following research questions:

- **RQ1:** What are the dominant ethical challenges associated with the clinical deployment of AI in medical imaging?
- **RQ2:** How do national and international governance frameworks address issues such as accountability, transparency, and patient rights in AI-driven diagnostics?
- **RQ3:** What practical recommendations can be synthesised to support the integration of AI based on ethical principles and policy within the Australian healthcare system and beyond?

These questions form the backbone of the literature selection, coding taxonomy, and thematic analysis presented in the subsequent sections.

### 2.2. Search Strategy and Data Sources

An integrative multiphase search strategy was implemented to capture the interdisciplinary literature at the intersection of artificial intelligence, medical imaging, ethics, and health policy. The search covered publications from January 2018 to April 2025 and was designed to capture both foundational and contemporary contributions relevant to the evolving AI landscape. Recognising the rapid advancement of AI technologies and regulatory frameworks, this timeframe provided a balance between historical context and current relevance. All database searches were conducted and finalised in April 2025, after which the retrieved records were exported for screening, eligibility assessment, and thematic synthesis.

The search was carried out in three iterative phases to maximise coverage and minimise omission bias.

1. **Systematic Database Search:** Core academic repository was queried using controlled vocabulary and Boolean keyword combinations. The databases included PubMed/MEDLINE, ACM Digital Library, IEEE Xplore, SpringerLink, Elsevier ScienceDirect, and Google Scholar. PubMed/MEDLINE was included to ensure comprehensive coverage of biomedical, clinical, and healthcare-focused literature, while the remaining databases captured technical, interdisciplinary, and policy-oriented research. Keywords were selected to reflect both technical and normative domains (e.g., ‘AI in radiology’, ‘algorithmic bias’, ‘SaMD regulation’, ‘explainability in diagnostics’), including trust-related terms such as ‘trust’, ‘adoption’, ‘acceptance’, ‘calibration’, and ‘clinician–AI concordance’.
2. **Snowball Sampling:** To ensure the inclusion of high impact and often cited studies, backward snowballing (mining reference lists of key papers) and forward snowballing (identifying cited works) were used. This phase enriched the dataset with both seminal and emergent literature not captured through standard indexing.
3. **Gray Literature and Policy Integration:** In recognition of the growing influence of regulatory bodies and think tanks in shaping AI governance, authoritative non-academic sources were incorporated. These included white papers and guidance documents from the World Health Organisation (WHO), the OECD, the European Commission, the US Food and Drug Administration (FDA), and the Therapeutic Goods Administration of Australia (TGA), among others.

This multisource, temporally inclusive search protocol ensured that the resulting literature corpus was comprehensive and representative of the diverse scholarly, regulatory, and clinical perspectives surrounding AI in medical imaging.

### 2.3. Inclusion and Exclusion Criteria

To maintain thematic integrity and ensure academic rigor, we applied clear eligibility parameters during the screening process. These criteria were designed to include interdisciplinary perspectives in ethics, governance, and AI applications in healthcare. The primary evidence corpus comprised sources published between January 2018 and April 2025. Earlier publications cited throughout the manuscript were used only to provide foundational, historical, conceptual, or regulatory context and were not included in the formal evidence synthesis.

- **Inclusion Criteria:** Peer-reviewed journal articles, regulatory documents, white papers, and institutional reports published between January 2018 and April 2025. The included works addressed the ethical, legal, governance, or sociotechnical dimensions of AI in medical diagnostics, with specific relevance to imaging modalities.
- **Exclusion Criteria:** Technical AI research lacking ethical, clinical, or policy considerations; publications not related to healthcare domains (e.g., industrial or military AI); and non-English-language sources. English-language publications were included to ensure consistent screening, interpretation, and thematic synthesis across a large interdisciplinary body of literature spanning technical, clinical, ethical, legal, and policy domains. Given resource and translation constraints, non-English publications were excluded. While this may have resulted in the omission of some relevant studies, English-language sources were considered sufficient to capture major international developments in AI ethics, governance, and diagnostic imaging.

### 2.4. Keyword Matrix and Search Query Design

The search strategy was guided by a structured matrix of keyword clusters aligned with the conceptual pillars of the study. This matrix ensured comprehensive semantic coverage and supported consistent query formulation across diverse databases.

- **AI Application Domains:** “medical imaging”, “radiology AI”, “diagnostic automation”, “deep learning in healthcare”, “computer-aided diagnosis”.
- **Ethical Constructs:** “algorithmic bias”, “AI fairness”, “explainability”, “accountability in AI”, “clinical transparency”, “informed consent”.
- **Governance and Legal Constructs:** “AI regulation in healthcare”, “FDA AI policy”, “EU AI Act 2024 healthcare”, “SaMD compliance”, “data protection law”, “health AI governance”.

Database-specific syntax (e.g., proximity operators, Boolean logic) was used to tailor query execution. Synonyms and regional variations (e.g., “ethics” and “bioethics”, “regulation” and “compliance”) were included to ensure cross-disciplinary relevance.

### 2.5. Screening and Classification Protocol

All retrieved sources were imported into a central reference manager and screened at the level of title, abstract, and full text. Eligibility was assessed by two independent reviewers. Disagreements regarding study eligibility or thematic classification were resolved through discussion and reviewer consensus. During title and abstract screening, records were assessed against predefined eligibility criteria to determine their relevance to AI-enabled diagnostic imaging and associated ethical, governance, regulatory, clinical, or implementation considerations. Potentially eligible records subsequently underwent full-text review. Sources were included when they provided substantive discussion of ethical, legal, governance, regulatory, technical, clinical, or sociotechnical issues relevant to the development, deployment, oversight, or responsible implementation of AI in diagnostic imaging. The selected articles were coded using a tripartite classification system:

- **Ethics-Focused:** Literature emphasising bias mitigation, transparency, patient autonomy, or clinician-patient trust.
- **Governance-Orientated:** Works discussing regulatory instruments, legal liability, institutional policy, or compliance frameworks.
- **Clinical Utility:** Studies examining the impact of AI in the real world on diagnostic performance, efficiency, or clinical workflows.

This taxonomy facilitated cross-sectional synthesis and enabled the triangulation of ethical principles with policy and practice dimensions.

### 2.6. Thematic Domain Classification

Following full-text review, all included sources were categorised according to their primary thematic focus. An inductive thematic classification process was used to organise the literature into four overarching domains: (1) Ethical Considerations, (2) Broader Healthcare Applications, (3) Diagnostic Imaging Implementation, and (4) Governance, Policy, and Education.

Studies examining fairness, transparency, accountability, privacy, trust, informed consent, and related ethical issues were classified under Ethical Considerations. Publications exploring AI applications across broader healthcare contexts beyond diagnostic imaging were categorised as Broader Healthcare Applications. Studies focusing on the development, validation, deployment, or clinical integration of AI systems within diagnostic imaging workflows were grouped under Diagnostic Imaging Implementation. Sources primarily addressing regulatory frameworks, governance mechanisms, policy development, professional guidance, workforce preparedness, or educational considerations were assigned to Governance, Policy, and Education.

Where publications addressed multiple thematic areas, classification was based on the dominant focus identified during full-text review. Any uncertainties regarding thematic assignment were discussed among the review team and resolved through consensus. Thematic classification was undertaken through an iterative coding and synthesis process. Recurring concepts identified across the included sources were initially coded and subsequently grouped into higher-order thematic categories. These concepts included fairness and bias, explainability and transparency, accountability and oversight, privacy and data governance, regulatory alignment, clinical implementation, stakeholder trust, and post-deployment monitoring. The resulting thematic structure informed both the synthesis of governance challenges presented throughout the review and the development of the proposed conceptual governance framework.

### 2.7. Study Selection Summary

The database search initially yielded 642 records. After removal of duplicates (98), 544 titles and abstracts were screened. Full-text assessment was conducted for 213 articles, resulting in 156 studies included in the final synthesis.

Exclusions at the full-text stage were primarily due to: (i) studies focused solely on algorithmic performance without ethical or governance analysis, (ii) AI applications outside diagnostic imaging, (iii) non-peer-reviewed sources, and (iv) insufficient methodological transparency.

The final data set consisted of 156 sources, including peer-reviewed publications, regulatory documents, policy reports, and professional guidance materials, all of which were subjected to thematic coding and interdisciplinary synthesis. To enhance methodological rigour, study selection and eligibility assessments were conducted collaboratively by all authors using the predefined inclusion and exclusion criteria. Any uncertainties regarding article eligibility, classification, or thematic relevance were discussed among the review team and resolved through consensus. This adjudication process helped ensure consistency and transparency throughout the screening, selection, and synthesis stages of the review.

### 2.8. Thematic Synthesis Methodology

Following study selection, the included sources underwent full-text review, thematic coding, and interdisciplinary synthesis. A thematic synthesis approach was employed, allowing identification of cross-domain patterns, emergent policy gaps, and unresolved ethical concerns. Each source was assessed for the presence of normative frameworks (e.g., fairness principles, transparency standards), empirical validation (e.g., model evaluation in diverse populations), and governance positioning (e.g., regulatory stance, oversight mechanisms). These sources were systematically classified into four thematic domains: ethical considerations, broader healthcare applications, implementation of diagnostic imaging, and governance, policy, and education.

In addition, where reported in the literature, indicators related to trust and real-world adoption, including clinician uptake, patient acceptance, calibration reliability, and concordance with expert judgement, were considered as part of the thematic synthesis. A supplementary data extraction matrix listing the included sources, evidence classifications, and thematic coding categories has been provided as Supplementary File S3 to support transparency, traceability, and reproducibility of the thematic synthesis.

### 2.9. Assessment of Evidence Types

Given the interdisciplinary nature of the included studies, a single quantitative risk-of-bias instrument was not applied. Instead, sources were categorised according to evidence type (empirical deployment study, clinical validation, technical method development, policy or regulatory guidance, and conceptual analysis).

Empirical deployment studies and regulatory documentation were prioritised when deriving governance implications, while conceptual and technical works informed ethical domain synthesis. This structured evidence categorisation strengthened interpretive consistency and supported alignment between proposed governance pillars and documented implementation challenges.

### 2.10. Methodological Considerations and Limitations

Several methodological limitations warrant consideration:

- **Language Scope:** The study included only sources in English, which may have excluded relevant regional insights.
- **Technological Rapid Evolution:** The review may not fully reflect the latest advances in foundation models and real-time AI deployment.
- **Stakeholder Diversity:** Perspectives from patients, frontline clinicians, and minority populations were limited in the peer-reviewed literature and were supplemented by grey literature and institutional reports where feasible.

The review was not prospectively registered because it was designed as a PRISMA-ScR-informed systematic survey focused on thematic synthesis, governance analysis, and interdisciplinary policy integration rather than quantitative intervention evaluation. Given the interdisciplinary and predominantly conceptual nature of the included literature, quantitative meta-analysis and formal effect-size synthesis were not appropriate.

Despite these limitations, the review methodology aligns with best practices in systematic and scoping reviews and reflects the interdisciplinary depth required to interrogate both the technical and normative dimensions of AI in medical imaging.

### 2.11. Example Search Strategy

To strengthen methodological transparency and reproducibility, an example Boolean search strategy used during the PRISMA-ScR-informed systematic survey process is provided below Box 1. Search queries were iteratively refined across databases to account for differences in indexing systems, controlled vocabularies, and platform-specific search functionalities. Complete database-specific search strategies, including database-adapted Boolean search strings, are provided in Supplementary Material S1 to support methodological transparency and reproducibility.

Additional keyword variations and database-specific adaptations were applied where appropriate to maximise interdisciplinary coverage across technical, ethical, regulatory, and clinical literature sources.

Box 1 **Example Database: PubMed/MEDLINE.**

(“artificial intelligence” OR “machine learning” OR “deep learning” OR “large language models” OR LLM OR CNN)

AND

(“medical imaging” OR radiology OR diagnostics OR “diagnostic imaging”)

AND

(ethics OR governance OR fairness OR explainability OR transparency OR accountability OR privacy)

AND

(healthcare OR clinical OR hospital)

### 2.12. Synthesis Summary and Methodological Contribution

The structured methodology adopted in this survey enables a comprehensive and rigorous interrogation of ethical and governance challenges in AI-enabled diagnostic imaging. By integrating multi-source data, a clear classification scheme, and a robust synthesis strategy, this section forms the empirical foundation for the findings presented in Section 3.8. It directly supports the development of ethically grounded and policy-aware recommendations for the implementation of AI in healthcare. Importantly, this survey does not treat the reviewed literature as a collection of independent contributions, but as an interconnected body of work that reflects shared assumptions, recurring governance gaps, and divergent regulatory approaches. The synthesis presented in the following sections therefore emphasises cross-study comparison, thematic convergence, and points of tension between technical innovation and ethical oversight, rather than isolated summaries of individual publications.

## 3. Survey Results

The results presented in this section come from a comprehensive synthesis of scholarly and regulatory sources identified through a methodologically rigorous literature review protocol (see Section 2). The findings are mapped to the research questions described in Section 2 and provide a thematic distillation of current practices, ethical concerns, governance models, and stakeholder perspectives on AI integration in medical imaging.

For clarity, the results are presented in six linked parts: (1) search and selection outcomes, (2) characteristics of the included sources, (3) contemporary AI-enabled diagnostic imaging workflows, (4) ethical and governance risks identified across the literature, (5) stakeholder perspectives and trust-related findings, and (6) emerging governance models and synthesis of key findings. This structure distinguishes descriptive search outcomes from thematic findings and framework-relevant synthesis.

### 3.1. Search and Selection Summary

**Figure 4:**
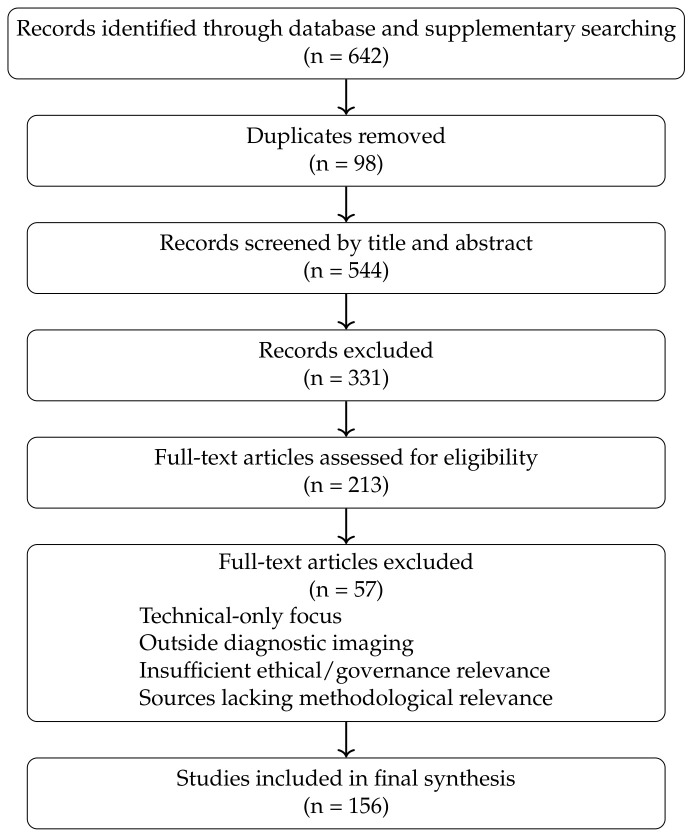
PRISMA-ScR flow diagram illustrating the study selection process for the systematic survey of sources published between 2018 and 2025.

The literature search and screening process identified 642 records across the selected databases and supplementary sources. After removal of 98 duplicate records, 544 records underwent title and abstract screening. Following eligibility assessment, 213 full-text articles and documents were reviewed, resulting in the inclusion of 156 sources in the final synthesis. The included evidence comprised peer-reviewed publications, regulatory documents, policy reports, and professional guidance materials. The study selection process is summarised in Figure 4.

### 3.2. Characteristics of Included Sources

The final dataset comprised 156 sources published between 2018 and 2025, reflecting the interdisciplinary nature of AI governance in diagnostic imaging. Sources included peer-reviewed journal articles, regulatory documents, policy reports, professional guidance materials, and institutional publications. For thematic synthesis, the included sources were categorised into four primary domains: ethical considerations (52 sources), broader healthcare applications (57 sources), diagnostic imaging implementation (43 sources), and governance, policy, and education (4 sources). Although some sources addressed multiple thematic areas, each was assigned to the domain representing its primary analytical focus. The distribution of included sources is presented in Table 1. To support transparent interpretation of the findings, evidence sources were analysed according to their primary contribution to the review. Empirical studies were used to identify reported implementation challenges, clinical outcomes, and deployment experiences. Regulatory documents, policy reports, and professional guidance materials informed the analysis of governance mechanisms, regulatory approaches, and accountability structures. Conceptual and normative publications primarily contributed to the synthesis of ethical principles, theoretical concerns, and emerging governance considerations. This evidence classification was maintained throughout the synthesis to ensure that policy recommendations and governance implications were interpreted in the context of the underlying evidence type.

### 3.3. Contemporary AI Workflows in Medical Imaging

#### 3.3.1. AI-Integrated Diagnostic Pipelines

Analysis of the reviewed literature indicated that AI integration in diagnostic imaging most commonly occurs across four interconnected workflow stages: (i) *image acquisition*, (ii) *data preprocessing and harmonisation*, (iii) *AI model inference*, and (iv) *clinical decision support* [12,70]. Across the included studies, this modular workflow architecture emerged as the dominant model for incorporating AI into radiological practice. The literature further showed that convolutional neural networks (CNNs), transformers, and more recently large language models (LLMs) were the technologies most frequently applied within these workflows. These systems were commonly used for tasks including anomaly detection, segmentation, disease classification, risk stratification, and automated reporting.

#### 3.3.2. Data Harmonisation and Preprocessing Techniques

The reviewed literature consistently identified data harmonisation and preprocessing as critical prerequisites for reliable AI performance across clinical settings. Commonly reported approaches included contrast normalisation, artefact removal, and anatomical labelling [44,70,109,110,111,112,113,114,115,116,117]. These steps reduce scanner variability and improve generalisability across institutions, key to multicentre AI deployment.

#### 3.3.3. AI Inference and Clinical Integration

Across the included studies, AI inference systems were most commonly applied to disease classification, risk stratification, and treatment prioritisation tasks. Several studies also reported increasing use of multimodal approaches [13,101]. LLMs are increasingly used to synthesise multimodal input (e.g., images + EHR notes), generating clinically actionable insights. However, this introduces complexities in interpretability and legal accountability [34,71].

#### 3.3.4. Systemic Feedback and Lifecycle Integration

The reviewed studies indicated a growing trend towards integrating AI systems with radiology infrastructures such as PACS and RIS. Feedback mechanisms from clinicians support continuous learning, but pose challenges regarding data provenance, system auditability, and over-reliance [10,14,112,142,144].

### 3.4. Ethical and Governance Risks Across Workflows

#### 3.4.1. Opacity and Interpretability Deficits

The reviewed literature consistently identified the black-box nature of deep learning systems as a prominent challenge, undermining clinician trust and the post hoc justification of AI decisions [70,73]. Studies further highlighted implications for patient consent, shared decision-making, and medico-legal responsibility [40,144].

#### 3.4.2. Bias and Underrepresentation

The reviewed studies consistently reported algorithmic bias arising from unbalanced or non-representative datasets [69,99]. Disparate performance in subgroups, particularly among under-represented populations and rare pathologies, risks perpetuating inequities in healthcare provision [11,26].

#### 3.4.3. Governance Fragmentation and Lifecycle Blind Spots

The reviewed literature identified a lack of consensus regarding risk classification, post-market surveillance, and model update mechanisms, resulting in persistent regulatory gaps [101,103]. Studies further indicated that few jurisdictions adequately address the dynamic nature of AI systems, particularly those capable of real-time learning, continual adaptation, or performance drift [70,107].

#### 3.4.4. Data Security and Privacy Exposure

The reviewed literature highlighted data security and privacy risks associated with the large volumes of sensitive health data required by AI systems, including concerns relating to reidentification, consent, and regulatory compliance [27,44]. Studies further indicated that although federated learning and encryption-based approaches offer potential mitigation pathways, their adoption in operational clinical environments remains limited [70].

### 3.5. Stakeholder Perspectives on Ethical AI

Stakeholders express various perceptions based on professional roles.

- **Clinicians and Radiologists:** Supportive of AI’s diagnostic potential but cautious due to trust and accountability concerns [13,149].
- **Patients:** Recognise benefits of speed and personalisation but express concern about the misuse of data and the opacity of decisions [11,41].
- **Developers:** Focus on technical efficacy and data access, often underestimating ethical downstream effects [46,74].

This misalignment requires the participation of multiple stakeholders in AI design and deployment [73,97].

### 3.6. Trust as a Measurable Dimension of Clinical AI Governance

Although trust is frequently invoked in discussions of artificial intelligence (AI) governance in healthcare, it is often treated as a broad normative aspiration rather than as a measurable implementation outcome. In AI-enabled medical imaging, this creates an important translational gap. A system may satisfy high-level expectations relating to fairness, transparency, privacy, and accountability, yet still fail to achieve meaningful clinical adoption if clinicians do not trust its outputs, patients do not accept its use, or institutions cannot verify its reliability under real-world conditions [41,109,117]. Trust should therefore be understood not only as an ethical objective, but also as an operational dimension of governance that can be assessed through empirical indicators across the AI lifecycle.

Within diagnostic imaging, trust is shaped by both technical and sociotechnical conditions. At the technical level, clinicians are more likely to rely on AI systems when predicted probabilities are well calibrated, when performance remains stable across settings, and when outputs are consistent with expert judgement in clinically meaningful tasks. At the sociotechnical level, trust is influenced by interpretability, accountability, and stakeholder understanding of AI-supported decisions [40,74,116]. These factors have been widely identified as barriers to trustworthy deployment, particularly where systems remain opaque or poorly integrated into clinical workflows.

To address this gap, trust can be integrated into lifecycle evaluation through a set of measurable indicators that connect governance principles to observable outcomes. During model development and validation, calibration measures can assess whether predicted probabilities align with true outcome frequencies, thereby indicating whether the system communicates confidence in a clinically reliable manner. During validation and deployment, radiologist–AI concordance can provide an empirical signal of alignment between model outputs and expert interpretation. During clinical use and post-deployment monitoring, patient acceptance rates, clinician uptake patterns, and override behaviour can help determine whether the system is trusted in practice rather than merely approved in principle [89,109].

These indicators should not be interpreted in isolation. Calibration without fairness may reinforce inequitable trust, while concordance without interpretability may conceal automation bias. For this reason, trust should be conceptualised as a cross-cutting governance outcome emerging from the interaction of ethical and regulatory dimensions identified in this review, including fairness, transparency, accountability, and privacy [48,69]. Trust therefore functions as an evaluative layer that determines whether governance mechanisms are effective in real-world clinical contexts.

From a governance perspective, integrating trust metrics strengthens the practical relevance of policy recommendations. Rather than recommending transparency, fairness auditing, or post-market monitoring only as normative safeguards, institutions can attach measurable trust-related indicators to each stage of implementation. This enables a shift from principle-based governance toward evidence-informed governance capable of evaluating whether AI systems are not only technically functional and legally compliant, but also clinically credible and socially legitimate.

In this sense, trust quantification does not replace ethical governance; rather, it provides the missing bridge between governance design and real-world adoption. For AI in medical imaging to be deployed responsibly, governance frameworks must demonstrate not only what safeguards exist, but also whether those safeguards produce trustworthiness that is visible, measurable, and sustainable in practice.

### 3.7. Emerging Governance Models

Several jurisdictions have introduced AI-specific regulatory or guidance frameworks. The European Union’s AI Act classifies medical AI as high-risk and mandates conformity assessments and post-market monitoring. The U.S. FDA regulates AI-enabled medical devices through Software as a Medical Device (SaMD) pathways, increasingly incorporating adaptive oversight mechanisms. The United Kingdom and Canada have emphasised lifecycle governance and algorithmic transparency, while China has introduced targeted algorithm regulations for clinical and consumer-facing systems.

Despite structural differences, these models share common themes: risk-based classification, documentation requirements, and increasing attention to post-deployment monitoring. However, operationalisation of adaptive oversight for continuously learning systems remains underdeveloped across jurisdictions. The European Union (EU), United States (US), and Australia were selected for detailed analysis because they represent three influential and contrasting approaches to AI governance in healthcare. The EU provides a comprehensive risk-based regulatory framework through the AI Act, the US adopts a sector-specific and lifecycle-oriented regulatory model centred on FDA oversight, and Australia represents an emerging governance environment that combines existing regulatory mechanisms with voluntary ethical principles. Together, these jurisdictions offer a useful comparison of mature, evolving, and hybrid governance approaches, while providing insights relevant to the responsible implementation of AI in healthcare systems internationally.

#### 3.7.1. The European Union (EU)

The EU leads globally with its proposed *AI Act 2024*, a landmark regulatory initiative that introduces a risk-based classification framework for artificial intelligence applications. In this approach, AI systems are classified into unacceptable, high, limited, and minimal risk levels, with healthcare applications—especially those that involve diagnosis or clinical decision support—typically falling into the high-risk category [13,107]. This classification triggers stringent requirements, including mandatory conformity assessments, algorithmic transparency obligations, data quality standards, cybersecurity safeguards, and provisions for human oversight and intervention [1,2,3,4,5,6,7,8,9,10,15,26,59,60,64,69,126,127,128,129,130,131,132].

A defining feature of the EU AI Act 2024 is its emphasis on upstream risk mitigation. High-risk systems must comply with technical documentation rules that enable traceability and reproducibility, including logging mechanisms and lifecycle performance reporting. The Act also mandates post-market surveillance obligations and empowers national supervisory authorities with enforcement capabilities in EU member states [62,65]. This comprehensive framework represents a shift away from reactive and harm-based regulation to anticipatory governance based on system design, deployment context, and intended functionality [61,63]. However, implementation challenges persist. The different levels of digital maturity, regulatory capacity, and healthcare infrastructure in EU member states threaten to create inconsistencies in enforcement and interpretation [67,68]. Furthermore, the AI Act currently lacks explicit mechanisms for dealing with adaptive or self-improving algorithms, such as continual learning systems or foundation models, that evolve after market approval. This regulatory gap raises concerns about auditability, accountability, and model drift in real-world clinical settings [72,107,108].

In addition, the Act does not yet provide detailed technical criteria for explainability, fairness, or robustness, leaving open questions about how these principles should be operationalised in clinical workflows [34,73]. Stakeholders have also expressed concerns about the compliance costs of smaller AI developers and healthcare institutions, who may lack the legal and technical resources required to meet these obligations [70,100]. Despite these limitations, the EU AI Act 2024 sets an important precedent in establishing a unified, legally binding framework for ethical AI governance. Its influence is already observable in other jurisdictions, prompting parallel efforts in Canada, Brazil, and parts of Southeast Asia to design risk-based AI regulation [11,13,19,44,70]. If effectively implemented and continuously updated to account for technological advancements, the Act could serve as a global benchmark for responsible AI deployment in healthcare and beyond.

#### 3.7.2. The United States (US)

The US adopts a lifecycle-centered approach to AI governance in healthcare, regulated primarily through the Food and Drug Administration’s (FDA) *Software as a Medical Device* (*SaMD*) framework. This model emphasises real-world performance monitoring, benefit-risk assessments, and update traceability throughout the product lifecycle. Rather than approving AI systems based solely on static pre-market submissions, the FDA encourages adaptive oversight through its *AI*/*ML-Based SaMD Action Plan*, which lays out principles for algorithm transparency, performance evaluation, and a total product lifecycle (TPLC) approach [16,17,103,104]. Central to this framework is the requirement for manufacturers to demonstrate analytical and clinical validation through real-world evidence (RWE), including post-market surveillance data, to support safety and effectiveness in various clinical environments [19,20]. The model also promotes transparency around algorithmic changes, particularly for systems employing machine learning (ML) or deep learning (DL) techniques that may evolve after deployment. The concept of a “Predetermined Change Control Plan” (PCCP) has been introduced to allow for regulated, pre-approved updates without the need for full resubmission, an essential mechanism for supporting continuous learning systems [24,25].

Despite these innovations, several regulatory gaps remain. The FDA currently lacks a dedicated AI-specific regulatory category different from traditional SaMDs, which can lead to interpretive inconsistencies, especially in cases involving generative models or complex multimodal inference engines [18,22]. This ambiguity complicates clinical integration, particularly when AI systems serve as autonomous agents in classification, diagnosis, or therapeutic decision-making [21,23]. Furthermore, concerns persist around the scalability and timeliness of FDA approvals, with critiques citing long review timelines and high evidentiary burdens that disproportionately affect smaller developers and startups [17,23]. The voluntary nature of some transparency recommendations, such as sharing model performance between demographic subgroups or disclosing the explainability limitations, also raises concerns about enforceability and accountability [16,24].

Unlike the centralised approach of the European Union, the US relies on a more fragmented and market-driven regulatory environment, often mediated by state laws, private sector ethics boards, and institutional review bodies. This fragmentation leads to variable enforcement between jurisdictions and institutions, undermining consistent ethical safeguards [19,25]. Despite these challenges, the FDA’s iterative evidence-based model holds promise for aligning innovation with safety and efficacy, particularly if supported by clearer federal legislation that examines the unique risks posed by adaptive AI systems. Ongoing legislative discussions, including proposals for an AI Bill of Rights and updates to HIPAA to account for AI-driven analytics, suggest a growing political will to strengthen regulatory clarity and public trust [17,21,23,25,103].

#### 3.7.3. Australia

Australia’s approach to AI governance in healthcare is characterised by a progressive, innovation-friendly orientation. The Australian Health Practitioner and Regulation Agency (AHPRA), Australia’s principal medical regulator, has embraced regulatory agility through the use of *regulatory policies* and pilot programmes that allow AI developers to test technologies in a controlled environment without facing full regulatory burdens. This has enabled experimentation with high-risk adaptive AI systems while mitigating immediate legal risk [3,6,70].

Complementing this technical flexibility, Australia has developed a set of voluntary ethical principles in AI under the auspices of the Department of Industry, Science and Resources. These principles emphasise values such as human-centered design, explainability, privacy, and accountability, in order to guide ethical AI development across sectors, including healthcare [4,46]. Furthermore, the Australian Alliance for Artificial Intelligence in Healthcare (AAAiH) has issued sector-specific frameworks to support AI adoption in clinical settings while maintaining transparency and public trust [5,10]. Despite these forward-leaning initiatives, significant regulatory gaps remain. One of the most pressing challenges is the lack of binding AI-specific legislation governing medical AI tools. Current approval pathways are still mainly grounded in traditional medical device frameworks, which are not well suited to address the unique risks associated with complex multimodal platforms [1,2,7]. This has led to inconsistency in the oversight practices between institutions and states, and some rely on institutional ethics boards or vendor self-assessment to evaluate AI systems, often without formalised national guidance [8,9]. This raises serious concerns about the long-term accountability, safety, and fairness of AI once integrated into routine clinical workflows. Current frameworks also do not assess demographic fairness, cultural safety, and accessibility for Aboriginal and Torres Strait Islander populations, exacerbating existing healthcare inequalities [26,70]. However, Australia is uniquely positioned to become a regional leader in AI governance. Its robust public health infrastructure and early engagement with AI ethics provide a foundation for crafting agile and context-sensitive regulatory frameworks [31,48,49,107].

Efforts such as the Australian Government’s Digital Health Strategy and the AI Action Plan signal the growing political recognition of these challenges. However, translating voluntary principles into enforceable sector-specific regulations will be critical to avoiding a governance vacuum as AI becomes ubiquitous in diagnostic imaging, triage, and clinical decision support systems [13,44,70,74]. In federated systems such as Australia, regulatory responsibilities are distributed across national and state authorities, creating additional coordination challenges [14,35,50,70].

Other jurisdictions, including the United Kingdom, Canada, and China, have introduced guidance frameworks emphasising transparency, lifecycle oversight, and algorithm accountability, though operational standards for adaptive systems remain in development [31,32,35,46,48,50].

### 3.8. The Need for a Structured AI Governance Model

Rapid integration of artificial intelligence (AI) into medical imaging has catalysed significant advances in diagnostic precision, workflow optimisation, and clinical decision support. However, these innovations also introduce profound ethical dilemmas and regulatory ambiguities that existing governance structures are ill-equipped to handle. In the absence of comprehensive oversight, the deployment of AI risks undermining public trust, exacerbating healthcare disparities, and enabling opaque decision-making that challenges traditional notions of clinical accountability.

As AI systems transition from static tools to adaptive, continuously learning entities, the regulatory demands placed on them increase exponentially. These technologies often operate in high-stakes environments, such as radiological triage, cancer screening, and surgical planning, where the cost of error can be life-changing. Moreover, the “black box” nature of many deep learning architectures complicates transparency and explainability, two essential prerequisites for ethical and safe implementation. Consequently, governance must evolve from fragmented, reactive measures into a proactive, lifecycle-aware framework that can anticipate and mitigate both known and emerging risks.

A structured and context-sensitive governance model for AI in Australian medical imaging should reflect not only international best practices but also the unique sociotechnical, cultural and legal dimensions of the local healthcare system. Drawing on comparative insights from global frameworks and recent regulatory experiments, such a model must rest on the following foundational pillars [6,13,70,101,103,104]:

- **Regulatory Standardisation:** AI governance must be codified through enforceable sector-specific legislation. These laws should define approval pathways for medical AI tools, establish pre-market testing protocols, mandate real-world performance monitoring, and incorporate mechanisms for dynamic reevaluation, especially for models that evolve over time. Regulatory alignment with international norms (for example, the EU AI Act 2024 or the FDA’s SaMD framework) can facilitate global interoperability, but must be adapted to local contexts and ethical expectations.
- **Bias Mitigation and Equity Assurance:** Structural bias in training data, model architecture, and clinical deployment perpetuates health inequities, disproportionately affecting historically marginalised groups. Governance should require fairness audits, demographic performance stratification, and equity impact assessments throughout the AI lifecycle. In addition, funding mechanisms and incentives for data sharing must be created to support the inclusion of under-represented populations in clinical data sets.
- **Explainability and Clinical Transparency:** Regulatory bodies must enforce explainability as a legal requirement for high-risk AI systems. This includes mandates for interpretable output, model rationale reporting, and user-level documentation that clinicians and patients can understand. The explanation should extend beyond technical traceability to include meaningful and context-relevant communication that supports informed consent and shared decision making.
- **Accountability and Legal Clarity:** Legal responsibility for AI-induced harm must be clearly allocated throughout the AI value chain. This includes defining the liability between developers, healthcare providers, institutions, and vendors. Governance frameworks should require documentation of AI decision-making processes (e.g., audit trails), robust incident reporting mechanisms, and third-party validation of safety claims. Without these measures, accountability becomes diffused, hindering redress, and eroding public trust.
- **Adaptive and Participatory Oversight:** Given the pace of technological innovation, static regulatory models will quickly become obsolete. A future-proof governance model must incorporate mechanisms for continuous learning, stakeholder consultation, and adaptive policy revision. Public and professional involvement, through citizen juries, ethics boards, and clinical feedback loops, should be embedded into the oversight infrastructure to ensure that evolving standards reflect societal values and frontline realities.

This governance vision must be co-designed through interdisciplinary collaboration, involving not only clinicians and technologists, but also patients, ethicists, policymakers, and legal experts. Such inclusive design processes are crucial for ensuring that ethical safeguards are embedded from the outset rather than retrofitted after deployment. In addition, Australia is well positioned to contribute to leadership in this area by developing a hybrid governance model that bridges ethical principles with enforceable regulation, tailored to its healthcare infrastructure, policy landscape, and digital maturity.

Table 7 presents a comparative overview of emerging AI governance models across major jurisdictions and international organisations. The table highlights differences in regulatory approaches, enforceability, governance mechanisms, and implementation challenges, providing a structured basis for understanding how various healthcare systems are responding to ethical, legal, and operational challenges associated with AI-enabled medical technologies [46,70,103,104,107]. This comparison informs the development of the proposed governance framework by identifying common strengths, recurring limitations, and opportunities for regulatory harmonisation across healthcare AI governance systems.

Table 7 demonstrates that while regulatory approaches differ substantially across jurisdictions, common challenges persist regarding adaptive AI oversight, accountability mechanisms, transparency, and post-deployment governance.

### 3.9. Synthesis of Key Findings

Thematic synthesis of the 156 included sources revealed several recurring patterns across the AI lifecycle. Although technological capabilities continue to advance rapidly, the literature consistently identified ethical, governance, and implementation challenges that may limit safe, equitable, and trustworthy deployment in clinical practice. The principal findings are summarised below.

- AI workflows in medical imaging are increasingly modular and multimodal, but remain constrained by ongoing challenges relating to explainability, transparency, and accountability.
- Ethical risks, including algorithmic bias, opacity, and privacy exposure, are widely documented but remain insufficiently addressed in many real-world clinical deployments.
- Stakeholders express divergent priorities and expectations, underscoring the importance of participatory, interdisciplinary, and lifecycle-aware governance approaches.
- Regulatory strategies remain fragmented, predominantly premarket-oriented, and insufficiently equipped to support continuous oversight of adaptive and continuously learning AI systems.
- Trust emerged as a cross-cutting governance concern, with the literature highlighting the need for measurable indicators such as calibration reliability, clinician–AI concordance, patient acceptance, and real-world adoption to support responsible implementation.

These findings reinforce the need for robust, enforceable, and interdisciplinary governance frameworks capable of balancing innovation with trust, safety, accountability, and patient-centred care.

To support analytical synthesis across the reviewed literature, ethical and governance challenges were organised according to the AI system lifecycle. This lifecycle-centric taxonomy enables systematic comparison of risks, governance responses, and recurring limitations reported across studies, providing a structured foundation for the thematic analysis and governance recommendations presented in the following sections.

Rather than treating ethical challenges as isolated concerns, the taxonomy highlights how governance gaps recur and evolve across different stages of the AI lifecycle. This structure is used throughout the remainder of the survey to guide comparative analysis and to inform the development of governance recommendations.

## 4. Integrated Findings and Synthesis

The systematic analysis of 156 scholarly and regulatory sources highlights deeply embedded and intersecting ethical and governance challenges surrounding the adoption of Artificial Intelligence (AI) in medical imaging and diagnostics. Although AI-driven technologies demonstrate growing promise in improving diagnostic accuracy, workflow efficiency, and personalised care, significant unresolved vulnerabilities persist in multiple dimensions. These include enduring deficits in algorithmic transparency and interpretability, structural biases arising from unrepresentative training datasets that risk exacerbating healthcare disparities, insufficient privacy-preserving mechanisms for handling sensitive medical data, and fragmented regulatory frameworks ill-equipped to govern rapidly evolving AI systems. The increasing deployment of sophisticated AI architecture, such as foundation models, generative systems, large multimodal models, and continuously learning algorithms, further intensifies these challenges by introducing adaptive behaviours, real-time model drift, and accountability ambiguities that transcend the capabilities of existing governance structures. Despite emerging governance efforts in select jurisdictions, current regulatory models often remain reactive, pre-market focused, and lack enforceable lifecycle oversight capable of managing the complex sociotechnical risks posed by AI-enabled diagnostic systems in diverse clinical environments [70,72,101,122]. Algorithmic bias remains a central concern, as many AI models are trained in data sets that do not adequately represent diverse populations, leading to unequal diagnostic outcomes between demographic groups [23,69,98]. The opacity of deep learning systems, often described as “black-box” models, continues to undermine trust and informed clinical decision making, despite advances in explainability tools such as saliency maps and local surrogate models [25,73,126,127].

A cross-cutting finding of this review is that while trust is frequently invoked as a central objective of ethical and regulatory frameworks for clinical AI, it is rarely operationalised through measurable or auditable indicators. Across the reviewed literature, trust is often assumed to emerge from model performance, transparency mechanisms, or regulatory compliance, yet few studies provide concrete approaches for evaluating trust in practice. Where measurable proxies are discussed, they tend to include factors such as model calibration reliability, clinician–AI concordance, patterns of clinical adoption or override behaviour, and patient acceptance in real-world contexts. This gap highlights a critical limitation in current governance approaches, where trust remains a normative aspiration rather than an empirically grounded dimension of AI system evaluation.

Legal accountability remains poorly defined in scenarios where autonomous AI systems contribute to clinical errors, raising unresolved questions about the allocation of responsibility among developers, vendors, and healthcare providers [100,103,145]. In parallel, data privacy concerns persist, driven by the scale and sensitivity of medical data aggregated for AI training. Existing consent models and anonymisation frameworks often lag behind technical developments, which requires more robust privacy preservation approaches, such as federated learning and secure multiparty computation [27,44]. At the governance level, regulatory fragmentation remains a global obstacle, with divergent standards and inconsistent oversight mechanisms between jurisdictions such as the FDA (USA), EMA (Europe), and TGA (Australia) [45,104,146]. Although some progress has been made toward developing international regulatory frameworks, these efforts often struggle to keep up with the rapidly evolving AI capabilities [46,70,77]. Crucially, these findings highlight a growing temporal gap between emerging ethical challenges and the slower institutional development of governance capacity. This is particularly relevant in the Australian healthcare context, where AI-driven imaging technologies are advancing, but governance mechanisms are still in the early stages [79,101]. Addressing these gaps requires not only ongoing interdisciplinary dialogue, but the formulation of adaptive, context-sensitive policy frameworks that prioritise fairness auditing, transparent accountability, and participatory oversight structures [31,86,108].

**Figure 5:**
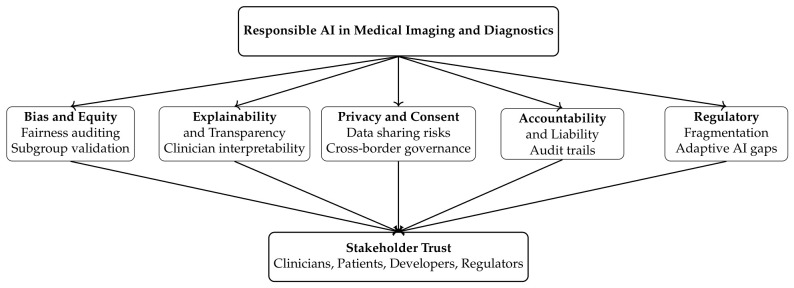
Conceptual synthesis of the principal ethical and governance challenges identified across the reviewed literature and their collective influence on stakeholder trust and the responsible implementation of AI in medical imaging and diagnostics.

To facilitate interpretation of the integrated findings, Figure 5 presents a conceptual synthesis of the principal ethical and governance challenges identified across the reviewed literature. The figure illustrates how recurring concerns relating to bias and equity, explainability and transparency, privacy and consent, accountability and liability, and regulatory fragmentation collectively influence stakeholder trust and the responsible implementation of AI in medical imaging and diagnostics.

Figure 5 illustrates that stakeholder trust emerges from the interaction of multiple ethical and governance domains rather than any single challenge in isolation, reinforcing the need for integrated lifecycle governance approaches.

### 4.1. AI Ethics vs. Technical Safety: Conceptual Divergence

In the reviewed literature, the terms *ethics*, *governance*, and *safety* are often conflated, although they serve distinct functions. Technical safety relates to robustness and performance; ethics considers fairness, autonomy, and rights; and governance refers to policy structures that ensure ethical compliance [13,70,101]. This conceptual ambiguity often results in fragmented strategies that neglect the full scope of responsible AI integration. For example, explainability methods are frequently promoted for clinical acceptance, but insufficient attention is paid to their ethical implications in decision-making [24,34,73].

### 4.2. Data Privacy and Consent in AI Imaging Systems

AI models in medical imaging are predominantly trained on large datasets containing sensitive patient information. However, a significant portion of the literature omits or marginalises privacy-preserving mechanisms such as federated learning, differential privacy, or secure multiparty computation [27,44]. Moreover, while HIPAA and GDPR are cited as guiding standards, implementation in jurisdictions remains inconsistent [32,70]. The use of cloud-based systems further exacerbates these concerns, especially when data is stored and processed across international borders with varying legal protections [13,101].

### 4.3. Algorithmic Bias: The Unresolved Equity Challenge

Numerous articles reported bias in the output of the AI model due to the under-representation of minority populations in training data [69,98,99]. This includes disparities in image quality, scanner types, and annotation accuracy in geographies. Despite growing awareness, systematic fairness auditing and demographic performance benchmarking remain largely absent from commercial and academic systems. Some studies advocated for debiasing techniques, but these approaches lack consensus on metrics and standards [26,34,97].

### 4.4. Opacity and the Need for Explainable AI (XAI)

Deep learning models used in imaging diagnostics often function as black-box systems. This opacity undermines trust and obstructs the legal and clinical validation of AI decisions [71,72,127]. XAI techniques such as saliency maps, Shapley values, and counterfactual explanations have been proposed, yet they are inconsistently adopted and insufficiently evaluated in medical contexts [24,31,73]. The literature reveals a tension between model performance and interpretability, with most clinical stakeholders favouring transparency over marginal gains in accuracy.

### 4.5. Governance Models and Global Fragmentation

The governance of AI in medical imaging lacks uniformity between jurisdictions. Our analysis revealed divergent policy frameworks, ranging from voluntary ethical principles (e.g., Australia’s AI Ethics Principles) to formal regulatory instruments (e.g., the EU AI Act 2024, FDA SaMD) [70,104,107]. Table 7 summarises these frameworks. In particular, Australia currently lacks enforceable legislation specifically addressing AI in healthcare protocols, making legal harmonisation, standards development, and multi-stakeholder oversight an urgent need [46,101].

### 4.6. Clinical and Stakeholder Perceptions

Clinicians value the ability of AI to accelerate diagnosis and improve decision making, but express concerns about its reliability and the risk of deskilling [41,42,139]. Patients want faster and more accurate diagnoses, but remain sceptical of data misuse and lack of transparency [44,58]. Developers prioritise model accuracy and data access, but often overlook ethical design and regulatory fit. These perspectives underscore the misalignment between technical advancement and societal readiness [11,13,46,70,79,98,100,107,108,109,110,111,112,113,114,115,116,117,135,136,137,138].

### 4.7. Discussion

Our systematic survey identifies six critical and interlinked findings that reflect both thematic convergence and persistent fragmentation across ethical, technical, and governance domains in AI-driven medical imaging. These insights underscore the urgent need for a comprehensive and enforceable governance framework geared to national contexts like Australia, while informed by global best practices [71,72,73,76,77,78,79,80,81,82,83,84,85,86,87,88,89,90,91,92,93,94,95,96,118,119,120,121,122,123,124,125,133,134,140,141,150,151,152,153].

- **Conceptual Misalignment Between Ethics, Safety, and Governance.** Across jurisdictions, ethical concerns (for example, fairness, consent), technical safety (for example, robustness), and governance (for example, regulation, liability) are frequently conflated, resulting in misaligned priorities, patchwork accountability, and unclear deployment standards [13,69,70,101]. Lack of definitional clarity inhibits stakeholder collaboration and weakens policy coherence.
- **Privacy Gaps and Cross-Border Data Vulnerabilities.** Despite increasing use of cloud-based infrastructure and transnational training pipelines, privacy-preserving techniques (e.g., federated learning, homomorphic encryption) remain underused or poorly regulated [27,44,120]. International divergence in data protection laws further complicates cross-jurisdictional compliance and undermines trust in AI systems [11,13,32,70,98,100,107,108,135,136,137,138].
- **Systemic Algorithmic Bias and the Absence of Equity Auditing.** Disparities in model performance across demographic subgroups, often resulting from non-representative training data, persist as one of the most widely cited and least addressed concerns in the literature [26,69,99]. Most regulatory frameworks do not mandate fairness benchmarking, leaving bias mitigation dependent on voluntary developer practices [31,100,154].
- **Explainability Remains a Bottleneck Despite Technical Progress.** While saliency maps, SHAP/LIME, and counterfactual explanations offer interpretability pathways, their clinical utility and reliability remain contested. Moreover, explanation tools have not yet been standardised or required by most governance regimes, posing risks to informed consent, clinician trust, and medicolegal defence [34,72,73].
- **Regulatory Fragmentation and Lifecycle Blind Spots.** Most jurisdictions lack comprehensive and enforceable governance structures that cover the entire AI lifecycle, including real-time learning, adaptive updates, and post-deployment monitoring [103,107]. Table 7 highlights the spectrum of maturity in governance, with only the EU proposing legally binding lifecycle oversight through its AI Act [104].
- **Stakeholder Priorities are Misaligned and Under-represented.** Clinicians emphasise interpretability and workflow integration; patients prioritise consent and data control; and developers focus on precision and scalability, yet few governance models institutionalise mechanisms for participatory co-design [41,46,58,155]. This misalignment exacerbates socioethical disconnects and delays real-world adoption.

Collectively, these findings reaffirm the necessity of a structured and interoperable ethical governance model outlined in Section 1.4 that incorporates enforceable standards, equity auditing, explainability benchmarks, and multistakeholder oversight. This model is essential to ensure that AI innovations in Australian healthcare are not only technically robust but also ethically sound, legally accountable, and socially trustworthy [13,70,100,156].

An important implication of this review is that trust in clinical AI is predominantly treated as a normative or aspirational concept rather than a measurable property of system performance and governance. While ethical and regulatory frameworks consistently emphasise the importance of trust, they rarely provide mechanisms for assessing it across different stages of the AI lifecycle. This study highlights the need to conceptualise trust as an evaluative dimension that can be examined through indicators such as calibration reliability, clinician–AI concordance, patterns of adoption and override behaviour, and patient acceptance in real-world clinical settings. Embedding such measurable trust indicators within governance frameworks can strengthen policy relevance, support implementation readiness, and enable more accountable and transparent integration of AI in diagnostic practice.

### 4.8. Implications for Digital Health Systems

The findings underscore that AI-enabled diagnostic imaging should be governed as part of an integrated digital health ecosystem rather than as isolated medical devices. Governance mechanisms must therefore align with electronic health record interoperability standards, institutional risk management structures, and national digital health strategies.

Lifecycle monitoring, subgroup performance transparency, and adaptive regulatory pathways are not solely technical safeguards; they are institutional capabilities that determine whether digital health systems can sustain public trust and clinical reliability. Without coordinated system-level oversight, technological innovation risks outpacing governance maturity across interconnected digital infrastructures.

### 4.9. Transferability Beyond the Australian Context

Although the proposed conceptual governance framework is illustrated using the Australian regulatory environment, its structural components are intentionally jurisdiction-agnostic. The lifecycle-centric approach, bias-auditing mechanisms, subgroup-performance reporting, human-in-the-loop oversight, and post-deployment monitoring requirements can be adapted across federated and centralised health systems.

Jurisdiction-specific elements such as privacy statutes or medical device pathways may vary, but the underlying governance architecture remains applicable to digital health ecosystems facing similar challenges of regulatory fragmentation, adaptive AI systems, and cross-institutional data flows.

Although the framework is informed by Australian healthcare governance arrangements and regulatory mechanisms, its underlying governance principles are derived from a broader international evidence base, including guidance from the World Health Organization, OECD AI Principles, FDA Good Machine Learning Practice guidance, IMDRF recommendations, and the European Union AI Act. Consequently, the framework is intended as a transferable conceptual structure that can be adapted to different healthcare systems while incorporating jurisdiction-specific legal, regulatory, organisational, and cultural requirements.

## 5. Conclusions and Future Research Directions

The rapid integration of AI into diagnostic imaging presents not only technical innovation but also increasing governance complexity across digital health ecosystems. This review synthesised 156 studies to identify recurring ethical risks and regulatory gaps across the AI lifecycle. The reviewed literature consistently highlighted challenges relating to explainability, algorithmic bias, accountability, privacy protection, stakeholder inclusion, and limitations in existing regulatory approaches for adaptive AI systems.

These findings directly reflect the thematic patterns identified throughout the Results section, where recurring governance gaps, stakeholder concerns, lifecycle management limitations, trust-related challenges, and regulatory fragmentation emerged consistently across the reviewed evidence base. Building directly on the findings synthesised from the reviewed literature, our lifecycle-centric governance framework translates widely recognised ethical principles into practical oversight mechanisms spanning development, deployment, monitoring, and adaptive updating. Although illustrated through the Australian context, the framework is transferable to federated and multi-jurisdictional health systems facing similar governance challenges.

The governance framework and policy recommendations presented in this review represent the authors’ synthesis and interpretation of evidence derived from empirical studies, regulatory documents, policy reports, and conceptual analyses rather than direct findings reported uniformly across all included sources. As a literature-derived conceptual framework, the proposed governance framework provides a structured foundation for future implementation and evaluation. Future research should assess its applicability, usability, and effectiveness across diverse clinical, organisational, and regulatory settings.

Future digital health governance should prioritise post-deployment surveillance, subgroup performance transparency, adaptive regulatory mechanisms for continuously learning systems, and measurable trust indicators, including calibration reliability, clinician–AI concordance, adoption and override behaviour, patient acceptance, and post-deployment monitoring signals. Without such institutional readiness, technological advancement may outpace the safeguards required for equitable and trustworthy clinical AI integration.

### 5.1. Persistent Limitations Hindering Ethical AI Integration

Despite growing scholarly attention and emerging regulatory initiatives, several structural and interrelated limitations continue to hinder the ethical integration of AI in medical imaging.

- **Absence of Clinically Grounded Explainability Standards:** While explainable AI (XAI) has advanced methodologically, most current techniques remain insufficient for clinical decision-making settings. Tools such as saliency maps, Grad-CAM, SHAP, and counterfactuals often do not align with clinicians’ reasoning processes and risk communication needs. The lack of standardised explainability benchmarks perpetuates legal ambiguity around liability and undermines clinician trust.
- **Systemic Algorithmic Bias and Equity Gaps:** Persistent underrepresentation of minority populations and structurally marginalised communities in training data contributes to unequal diagnostic performance across demographic subgroups. Despite increased awareness, regulatory mandates for fairness auditing, demographic validation, and post-deployment equity monitoring remain limited across most jurisdictions.
- **Fragmented and Reactive Governance Architecture:** Global governance frameworks remain fragmented, with significant variability in regulatory maturity, enforceability, and risk stratification approaches. Most existing models focus heavily on premarket approval while offering limited mechanisms for ongoing oversight, model drift detection, and adaptive regulatory updates. Australia, despite its innovation-friendly ethos, exemplifies this governance gap through the absence of comprehensive AI-specific healthcare legislation.
- **Underdeveloped Privacy-Preserving Infrastructure:** Although privacy-preserving approaches such as federated learning, homomorphic encryption, and secure multiparty computation offer promising pathways to ethical data sharing, their operational implementation remains rare. Institutional interoperability, technical complexity, and resource disparities continue to impede broader adoption in clinical practice.
- **Weak Stakeholder Engagement and Participatory Oversight:** The development and deployment of AI systems in healthcare remain largely siloed within technical, regulatory, or commercial domains, with insufficient participation from patients, clinicians, and marginalised communities. Stakeholder consultations often occur late in the design process or operate in advisory roles without decision-making authority, undermining the legitimacy and social robustness of AI governance structures.

### 5.2. Strategic Research Priorities for Responsible AI Governance

Addressing these limitations requires a coordinated and multidisciplinary research agenda that extends beyond technical optimisation to ethically embedded innovation. Key research priorities include:

- **Co-Governance Framework Development:** Future research should focus on empirically grounded governance models that integrate regulators, developers, clinicians, patients, ethicists, and civil society into ongoing governance processes. These frameworks should support adaptive regulation, continuous auditing, and sector-specific accountability while balancing domestic legal requirements with international harmonisation efforts.
- **Clinically Relevant Fairness and Explainability Metrics:** Research should prioritise fairness metrics that extend beyond technical parity to incorporate clinical relevance, subgroup stratification, and context-sensitive outcome evaluation. Likewise, explainability tools should be assessed not only for transparency but also for their alignment with clinical reasoning and medicolegal requirements.
- **Institutionalisation of Participatory AI Design Practices:** Structured stakeholder inclusion methodologies should be developed to ensure that patients, marginalised groups, and frontline clinicians have meaningful input throughout the AI lifecycle, from problem formulation to post-market surveillance.
- **Ethical Evaluation of Privacy-Preserving AI Infrastructure:** Technical assessments of privacy-preserving technologies should be complemented by ethical evaluations examining real-world feasibility, institutional trustworthiness, and alignment with diverse data protection regimes.
- **Lifecycle Accountability and Post-Deployment Governance:** New governance mechanisms are needed to address post-market challenges, including ethical sunset clauses, adaptive decommissioning protocols, real-time auditing tools, and responsive regulatory triggers aligned with evolving system behaviour and clinical environments.

### 5.3. The Australian Context: A Normative Governance Imperative

For Australia, the ethical integration of AI into healthcare is at a regulatory crossroads. Although the Therapeutic Goods Administration (TGA) has demonstrated leadership through regulatory initiatives and voluntary ethics principles, the absence of enforceable AI-specific legislation remains a significant vulnerability as adaptive and generative AI systems enter clinical workflows. Continued reliance on institutional discretion, self-regulation, and fragmented pilot initiatives risks creating an uneven governance landscape characterised by inconsistent standards, limited post-market accountability, and unresolved liability concerns.

Australia’s strong healthcare system, research capability, and early engagement with AI ethics position it to pioneer a globally relevant governance model. Such a model should combine the following:

- **Legally enforceable sector-specific AI legislation**, incorporating risk-tiered approval pathways, mandatory fairness audits, real-world evidence generation, and adaptive post-market surveillance.
- **Codified explainability and accountability standards**, which require human-interpretable model rationales that support both clinical safety and patient autonomy in informed consent processes.
- **Participatory and culturally responsive governance structures**, ensuring that indigenous populations, rural communities, and marginalised stakeholders are actively included in the governance of AI tools deployed in their healthcare contexts.
- **Global interoperability aligned with international best practices**, drawing selectively from leading models such as the EU AI Act 2024, FDA SaMD frameworks and WHO ethical principles, while tailoring implementation to Australia’s unique sociotechnical ecosystem.

### 5.4. Toward a Governance Foundation for Ethical AI Innovation

Ultimately, trustworthy AI in healthcare is not solely a technical objective but also a governance and societal imperative. Without enforceable mechanisms that embed fairness, transparency, and accountability throughout the AI lifecycle, public trust and clinical confidence may be undermined.

Sustainable AI integration in diagnostic imaging therefore depends on strengthening digital health governance infrastructures capable of coordinating innovation, regulation, and clinical accountability across interconnected healthcare systems.

## Figures and Tables

**Table 1:** Distribution of Included Sources Across Thematic Domains and Relative Representation (2018–2025).

Thematic Domain	Sources (n)	Percentage (%)
Ethical Considerations	52	33.3
Broader Healthcare Applications	57	36.5
Diagnostic Imaging Implementation	43	27.6
Governance, Policy, and Education	4	2.6
**Total**	**156**	**100.0**

**Table 2:** Temporal Distribution of Included Sources Across Thematic Domains (2018–2025).

Year	Ethical Considerations	Broader Healthcare Applications	Diagnostic Imaging Implementation	Governance, Policy, Education	Integrated in This Study
2018	2	1	1	0	✓
2019	7	5	4	0	✓
2020	10	9	7	1	✓
2021	9	10	7	1	✓
2022	8	10	7	1	✓
2023	8	9	7	0	✓
2024	5	8	5	1	✓
2025	3	5	5	0	✓
**Total**	**52**	**57**	**43**	**4**	**✓ (All)**

**Table 3:** Solution Taxonomy of Technical and Governance Responses Across the AI Lifecycle, Including Validation Maturity and Implementation Limitations.

Solution Category	Representative Methods	Lifecycle Stage	Validation Level	Key Limitations
Bias Mitigation	Reweighting, adversarial debiasing, subgroup performance reporting	Development/Validation	Retrospective clinical testing common	Limited prospective deployment validation; dataset imbalance persists
Explainability (XAI)	SHAP, LIME, saliency maps, counterfactual explanations	Validation/Deployment	Simulated and retrospective studies predominant	Lack of standardised clinical interpretability metrics; uncertain impact on decision-making
Privacy-Preserving Learning	Federated learning, differential privacy, secure aggregation	Data acquisition/Training	Pilot and simulation studies	Trade-offs between privacy guarantees and diagnostic performance
Post-Deployment Monitoring	Performance dashboards, drift detection algorithms, audit logging	Deployment/Monitoring	Emerging implementation evidence	Sparse regulatory standardisation; unclear escalation protocols
Regulatory Governance Tools	SaMD approval pathways, post-market surveillance, PCCP frameworks	Deployment/Lifecycle update	Policy guidance evolving	Limited integration with adaptive and continuously learning systems

**Table 4:** Key Functional Modules and Associated Technologies in AI-Enabled Medical Imaging.

Module	Core Functions	Illustrative Technologies
Image Acquisition	Captures diagnostic scans from various modalities	CT, MRI, PET, X-ray
Preprocessing	Enhances image quality, reduces artifacts, standardises inputs	Denoising, normalisation, contrast harmonisation
AI Inference Engine	Analyses patterns, detects abnormalities, generates predictions	CNNs, Transformers, LLMs, radiomics
Explainability Module	Improves transparency and model interpretability	SHAP, LIME, saliency maps, counterfactuals
Privacy Layer	Secures sensitive data and protects patient identity	Federated learning, homomorphic encryption
Clinical Decision Support	Delivers actionable insights and integrates into workflows	PACS/RIS integration, triage, automated reporting
Feedback Loop	Enables continuous learning from radiologist input	Human-in-the-loop validation and retraining

**Table 5:** Lifecycle-Centric Taxonomy of Ethical and Governance Challenges, Governance Responses, and Trust Indicators in AI-Enabled Medical Imaging.

AI Lifecycle Stage	Dominant Ethical Risks	Commonly Proposed Governance Responses	Key Limitations Identified in the Literature	Illustrative Trust Indicators
Data Collection and Curation	Dataset bias, lack of representativeness, secondary data use without consent, privacy leakage	Data governance policies, consent frameworks, bias audits, de-identification standards	Limited patient involvement, weak enforcement mechanisms, inadequate coverage of minority populations	Dataset representativeness, demographic coverage, patient consent participation rates
Model Development and Training	Algorithmic bias amplification, opacity of decision logic, reproducibility concerns	Model documentation, explainability tools, interdisciplinary review committees	Limited clinical validation of explainability methods, lack of standardisation across institutions	Calibration error, confidence reliability, subgroup performance consistency
Validation and Regulatory Approval	Inadequate external validation, misalignment with clinical workflows, unclear accountability	Regulatory certification processes, clinical trials, performance benchmarking	Static approval models that fail to address adaptive or continuously learning systems	Radiologist–AI concordance, external validation stability across datasets
Clinical Deployment and Use	Automation bias, over-reliance on AI outputs, unclear clinician responsibility	Human-in-the-loop requirements, professional guidelines, role delineation protocols	Ambiguous liability allocation, variable uptake across clinical contexts	Clinician adoption rate, override frequency, real-world reliance patterns
Post-Deployment Monitoring and Adaptation	Model drift, performance degradation, governance gaps for system updates	Post-market surveillance, audit trails, continuous monitoring requirements	Limited regulatory guidance for adaptive systems, fragmented oversight responsibilities	Patient acceptance rates, incident-triggered review, longitudinal trust feedback trends

**Table 6:** Mapping of Governance Pillars to Evidence Signals in the Reviewed Literature (n = 156).

Governance Pillar	Evidence Signals in Literature	Prevalence	Observed Gaps
Equity and Bias Mitigation	Subgroup performance reporting, dataset representativeness analysis, fairness metrics	High	Limited prospective validation; underrepresentation of minority populations
Explainability and Transparency	Use of SHAP, LIME, saliency maps, counterfactual explanations	Moderate	Limited clinical validation and standardisation of interpretability metrics
Accountability and Oversight	Human-in-the-loop review, audit trails, regulatory documentation, clinical governance committees	Moderate	Unclear delineation of clinician vs. AI responsibility; limited post-market accountability mechanisms
Privacy-Preserving Infrastructure	Federated learning, differential privacy, secure multiparty computation	Emerging	Limited deployment evidence; trade-offs with model performance insufficiently evaluated
Adaptive Regulatory Alignment	Lifecycle monitoring proposals, post-deployment surveillance, change control protocols	Low–Moderate	Sparse empirical guidance for managing model drift and continual learning systems

**Table 7:** Comparative Overview of Emerging AI Governance Models in Healthcare Across Major Jurisdictions and International Organisations.

Jurisdiction/Body	Regulatory Model	Enforceability	Core Governance Features	Key Limitations and Gaps
European Union (EU)	AI Act (risk-based)	Legally binding	Conformity checks, transparency, data traceability, post-market surveillance	No standards for adaptive AI; country-level variation; high compliance burden
United States (FDA)	Lifecycle-centered (SaMD)	Medium (sectoral)	Total Product Lifecycle (TPLC), real-world evidence, model change protocols (PCCP)	No dedicated AI category; slow approvals; fragmented oversight
Australia (TGA)	Innovation + voluntary ethics	Low–Medium (hybrid)	Policies, AI Ethics Principles, pilot testing pathways	No binding AI law; weak post-market monitoring; fairness not enforced
United Kingdom (UK)	Sector-led soft governance	Low (principles-based)	MHRA oversight, explainability, public trust initiatives	No central AI body; unclear accountability; legal ambiguity
Canada (Health Canada)	Draft AI Act (AIDA)	Low (pending)	Bias audits, non-discrimination, clinical validation	Early stage; uncertain scalability; overlaps in jurisdiction
Singapore (PDPC)	Co-regulatory policies	Low (voluntary)	Explainability, robustness, flexible policies	No enforcement power; redress unclear for high-risk use
China (NHC/Cyber Law)	State-centered mandates	High (state-controlled)	Algorithm registration, performance audits, data localisation	Limited transparency; opaque approvals; weak civil oversight
Japan (PMDA)	Post-market focused	Medium	Algorithm traceability, safety-first regulation	Slow on LLMs and generative AI; limited stakeholder feedback
WHO/OECD	Normative guidance	None	Rights-based ethics, transparency, sustainability	Advisory only; no enforcement; limited uptake in LMICs

## Data Availability

No new data were created or analysed in this study. Data sharing is not applicable to this article.

## Supplementary Materials

The following supporting information can be downloaded at: https://www.mdpi.com/article/10.3390/healthcare14131975/s1, Supplementary File S1: PRISMA-ScR Reporting Checklist; Supplementary File S2: Database-Specific Search Strategies and Search Dates; Supplementary File S3: Included Studies and Thematic Classification Matrix (Excerpt).

## References

[1] Bi W.L., Hosny A., Schabath M.B., Giger M.L., Birkbak N.J., Mehrtash A., Allison T., Arnaout O., Abbosh C., Dunn I.F. (2019). Artificial intelligence in cancer imaging: Clinical challenges and applications. CA A Cancer J. Clin..

[2] Habli I., Lawton T., Porter Z. (2020). Artificial intelligence in health care: Accountability and safety. Bull. World Health Organ..

[3] Guo Y., Hao Z., Zhao S., Gong J., Yang F. (2020). Artificial intelligence in health care: Bibliometric analysis. J. Med. Internet Res..

[4] Jimma B.L. (2023). Artificial intelligence in healthcare: A bibliometric analysis. Telemat. Inform. Rep..

[5] Chen M., Decary M. (2020). Artificial intelligence in healthcare: An essential guide for health leaders. Healthcare Management Forum.

[6] Kumar P., Chauhan S., Awasthi L.K. (2023). Artificial intelligence in healthcare: Review, ethics, trust challenges & future research directions. Eng. Appl. Artif. Intell..

[7] Holmes J., Sacchi L., Bellazzi R. (2004). Artificial intelligence in medicine. Ann. R. Coll. Surg. Engl..

[8] Liu P.r., Lu L., Zhang J.y., Huo T.t., Liu S.x., Ye Z.w. (2021). Application of artificial intelligence in medicine: An overview. Curr. Med. Sci..

[9] Beam A.L., Drazen J.M., Kohane I.S., Leong T.Y., Manrai A.K., Rubin E.J. (2023). Artificial intelligence in medicine. N. Engl. J. Med..

[10] Farhud D.D., Zokaei S. (2021). Ethical issues of artificial intelligence in medicine and healthcare. Iran. J. Public Health.

[11] Bekbolatova M., Mayer J., Ong C.W., Toma M. (2024). Transformative Potential of AI in Healthcare: Definitions, Applications, and Navigating the Ethical Landscape and Public Perspectives. Healthcare.

[12] Weiner E.B., Dankwa-Mullan I., Nelson W.A., Hassanpour S. (2025). Ethical challenges and evolving strategies in the integration of artificial intelligence into clinical practice. PLoS Digit. Health.

[13] Najjar R. (2023). Redefining radiology: A review of artificial intelligence integration in medical imaging. Diagnostics.

[14] Ahmad Z., Rahim S., Zubair M., Abdul-Ghafar J. (2021). Artificial intelligence (AI) in medicine, current applications and future role with special emphasis on its potential and promise in pathology: Present and future impact, obstacles including costs and acceptance among pathologists, practical and philosophical considerations. A comprehensive review. Diagn. Pathol..

[15] Barragán-Montero A., Javaid U., Valdés G., Nguyen D., Desbordes P., Macq B., Willems S., Vandewinckele L., Holmström M., Löfman F. (2021). Artificial intelligence and machine learning for medical imaging: A technology review. Phys. Medica.

[16] Huynh E., Hosny A., Guthier C., Bitterman D.S., Petit S.F., Haas-Kogan D.A., Kann B., Aerts H.J., Mak R.H. (2020). Artificial intelligence in radiation oncology. Nat. Rev. Clin. Oncol..

[17] Thompson R.F., Valdes G., Fuller C.D., Carpenter C.M., Morin O., Aneja S., Lindsay W.D., Aerts H.J., Agrimson B., Deville C. (2018). Artificial intelligence in radiation oncology: A specialty-wide disruptive transformation?. Radiother. Oncol..

[18] Ahmed Z., Mohamed K., Zeeshan S., Dong X. (2020). Artificial intelligence with multi-functional machine learning platform development for better healthcare and precision medicine. Database.

[19] Vaisman A., Linder N., Lundin J., Orchanian-Cheff A., Coulibaly J.T., Ephraim R.K., Bogoch I.I. (2020). Artificial intelligence, diagnostic imaging and neglected tropical diseases: Ethical implications. Bull. World Health Organ..

[20] Petersson L., Larsson I., Nygren J.M., Nilsen P., Neher M., Reed J.E., Tyskbo D., Svedberg P. (2022). Challenges to implementing artificial intelligence in healthcare: A qualitative interview study with healthcare leaders in Sweden. BMC Health Serv. Res..

[21] Iqbal M.J., Javed Z., Sadia H., Qureshi I.A., Irshad A., Ahmed R., Malik K., Raza S., Abbas A., Pezzani R. (2021). Clinical applications of artificial intelligence and machine learning in cancer diagnosis: Looking into the future. Cancer Cell Int..

[22] Beil M., Proft I., Van Heerden D., Sviri S., Van Heerden P.V. (2019). Ethical considerations about artificial intelligence for prognostication in intensive care. Intensive Care Med. Exp..

[23] McCradden M.D., Joshi S., Mazwi M., Anderson J.A. (2020). Ethical limitations of algorithmic fairness solutions in health care machine learning. Lancet Digit. Health.

[24] Chen I.Y., Pierson E., Rose S., Joshi S., Ferryman K., Ghassemi M. (2021). Ethical machine learning in healthcare. Annu. Rev. Biomed. Data Sci..

[25] Rasheed K., Qayyum A., Ghaly M., Al-Fuqaha A., Razi A., Qadir J. (2022). Explainable, trustworthy, and ethical machine learning for healthcare: A survey. Comput. Biol. Med..

[26] Albahri A.S., Duhaim A.M., Fadhel M.A., Alnoor A., Baqer N.S., Alzubaidi L., Albahri O.S., Alamoodi A.H., Bai J., Salhi A. (2023). A systematic review of trustworthy and explainable artificial intelligence in healthcare: Assessment of quality, bias risk, and data fusion. Inf. Fusion.

[27] Kaissis G.A., Makowski M.R., Rückert D., Braren R.F. (2020). Secure, privacy-preserving and federated machine learning in medical imaging. Nat. Mach. Intell..

[28] Jobin A., Ienca M., Vayena E. (2019). The global landscape of AI ethics guidelines. Nat. Mach. Intell..

[29] Shaheen M.Y. (2021). Applications of Artificial Intelligence (AI) in healthcare: A review. Sci. Prepr..

[30] Sun Q., Akman A., Schuller B.W. (2025). Explainable artificial intelligence for medical applications: A review. ACM Trans. Comput. Healthc..

[31] Char D.S., Abràmoff M.D., Feudtner C. (2020). Identifying ethical considerations for machine learning healthcare applications. Am. J. Bioeth..

[32] Zhang J., Zhang Z.m. (2023). Ethics and governance of trustworthy medical artificial intelligence. BMC Med. Inform. Decis. Mak..

[33] Geis J.R., Brady A.P., Wu C.C., Spencer J., Ranschaert E., Jaremko J.L., Langer S.G., Borondy Kitts A., Birch J., Shields W.F. (2019). Ethics of artificial intelligence in radiology: Summary of the joint European and North American multisociety statement. Radiology.

[34] Amann J., Blasimme A., Vayena E., Frey D., Madai V.I., Consortium P. (2020). Explainability for artificial intelligence in healthcare: A multidisciplinary perspective. BMC Med. Inform. Decis. Mak..

[35] Goirand M., Austin E., Clay-Williams R. (2021). Implementing ethics in healthcare AI-based applications: A scoping review. Sci. Eng. Ethics.

[36] Recht M.P., Dewey M., Dreyer K., Langlotz C., Niessen W., Prainsack B., Smith J.J. (2020). Integrating artificial intelligence into the clinical practice of radiology: Challenges and recommendations. Eur. Radiol..

[37] Nichols J.A., Herbert Chan H.W., Baker M.A. (2019). Machine learning: Applications of artificial intelligence to imaging and diagnosis. Biophys. Rev..

[38] Sun T.Q., Medaglia R. (2019). Mapping the challenges of Artificial Intelligence in the public sector: Evidence from public healthcare. Gov. Inf. Q..

[39] Grunhut J., Marques O., Wyatt A.T. (2022). Needs, challenges, and applications of artificial intelligence in medical education curriculum. JMIR Med. Educ..

[40] Reyes M., Meier R., Pereira S., Silva C.A., Dahlweid F.M., Tengg-Kobligk H.v., Summers R.M., Wiest R. (2020). On the interpretability of artificial intelligence in radiology: Challenges and opportunities. Radiol. Artif. Intell..

[41] Chew H.S.J., Achananuparp P. (2022). Perceptions and needs of artificial intelligence in health care to increase adoption: Scoping review. J. Med. Internet Res..

[42] Laï M.C., Brian M., Mamzer M.F. (2020). Perceptions of artificial intelligence in healthcare: Findings from a qualitative survey study among actors in France. J. Transl. Med..

[43] Olczak J., Pavlopoulos J., Prijs J., Ijpma F.F., Doornberg J.N., Lundström C., Hedlund J., Gordon M. (2021). Presenting artificial intelligence, deep learning, and machine learning studies to clinicians and healthcare stakeholders: An introductory reference with a guideline and a Clinical AI Research (CAIR) checklist proposal. Acta Orthop..

[44] Khalid N., Qayyum A., Bilal M., Al-Fuqaha A., Qadir J. (2023). Privacy-preserving artificial intelligence in healthcare: Techniques and applications. Comput. Biol. Med..

[45] Larson D.B., Harvey H., Rubin D.L., Irani N., Tse J.R., Langlotz C.P. (2021). Regulatory frameworks for development and evaluation of artificial intelligence–based diagnostic imaging algorithms: Summary and recommendations. J. Am. Coll. Radiol..

[46] Bouderhem R. (2024). Shaping the future of AI in healthcare through ethics and governance. Humanit. Soc. Sci. Commun..

[47] Arnold M. (2021). Teasing out artificial intelligence in medicine: An ethical critique of artificial intelligence and machine learning in medicine. J. Bioethical Inq..

[48] Morley J., Machado C.C., Burr C., Cowls J., Joshi I., Taddeo M., Floridi L. (2020). The ethics of AI in health care: A mapping review. Soc. Sci. Med..

[49] Li F., Ruijs N., Lu Y. (2022). Ethics & AI: A systematic review on ethical concerns and related strategies for designing with AI in healthcare. AI.

[50] Siala H., Wang Y. (2022). SHIFTing artificial intelligence to be responsible in healthcare: A systematic review. Soc. Sci. Med..

[51] Ahuja A.S. (2019). The impact of artificial intelligence in medicine on the future role of the physician. PeerJ.

[52] Doraiswamy P.M., Blease C., Bodner K. (2020). Artificial intelligence and the future of psychiatry: Insights from a global physician survey. Artif. Intell. Med..

[53] Krittanawong C. (2018). The rise of artificial intelligence and the uncertain future for physicians. Eur. J. Intern. Med..

[54] Yan Y., Zhang J.W., Zang G.Y., Pu J. (2019). The primary use of artificial intelligence in cardiovascular diseases: What kind of potential role does artificial intelligence play in future medicine?. J. Geriatr. Cardiol. JGC.

[55] Grunhut J., Wyatt A.T., Marques O. (2021). Educating future physicians in artificial intelligence (AI): An integrative review and proposed changes. J. Med. Educ. Curric. Dev..

[56] Zuhair V., Babar A., Ali R., Oduoye M.O., Noor Z., Chris K., Okon I.I., Rehman L.U. (2024). Exploring the impact of artificial intelligence on global health and enhancing healthcare in developing nations. J. Prim. Care Community Health.

[57] Gala D., Behl H., Shah M., Makaryus A.N. (2024). The role of artificial intelligence in improving patient outcomes and future of healthcare delivery in cardiology: A narrative review of the literature. Healthcare.

[58] Blease C., Worthen A., Torous J. (2024). Psychiatrists’ experiences and opinions of generative artificial intelligence in mental healthcare: An online mixed methods survey. Psychiatry Res..

[59] Grzybowski A., Jin K., Wu H. (2024). Challenges of artificial intelligence in medicine and dermatology. Clin. Dermatol..

[60] Johnson M., Patel M., Phipps A., Van der Schaar M., Boulton D., Gibbs M. (2023). The potential and pitfalls of artificial intelligence in clinical pharmacology. CPT Pharmacomet. Syst. Pharmacol..

[61] Aung Y.Y., Wong D.C., Ting D.S. (2021). The promise of artificial intelligence: A review of the opportunities and challenges of artificial intelligence in healthcare. Br. Med. Bull..

[62] Wubineh B.Z., Deriba F.G., Woldeyohannis M.M. (2024). Exploring the opportunities and challenges of implementing artificial intelligence in healthcare: A systematic literature review. Urologic Oncology: Seminars and Original Investigations.

[63] Olawade D.B., David-Olawade A.C., Wada O.Z., Asaolu A.J., Adereni T., Ling J. (2024). Artificial intelligence in healthcare delivery: Prospects and pitfalls. J. Med. Surg. Public Health.

[64] Bhagat S.V., Kanyal D. (2024). Navigating the future: The transformative impact of artificial intelligence on hospital management—A comprehensive review. Cureus.

[65] Bellini V., Russo M., Domenichetti T., Panizzi M., Allai S., Bignami E.G. (2024). Artificial intelligence in operating room management. J. Med. Syst..

[66] Khang A. (2024). AI and IoT Technology and Applications for Smart Healthcare Systems.

[67] Chen X., Xie H., Tao X., Wang F.L., Leng M., Lei B. (2024). Artificial intelligence and multimodal data fusion for smart healthcare: Topic modeling and bibliometrics. Artif. Intell. Rev..

[68] Pradyumna G., Hegde R.B., Bommegowda K., Jan T., Naik G.R. (2024). Empowering healthcare with IoMT: Evolution, machine learning integration, security, and interoperability challenges. IEEE Access.

[69] Chen R.J., Wang J.J., Williamson D.F., Chen T.Y., Lipkova J., Lu M.Y., Sahai S., Mahmood F. (2023). Algorithmic fairness in artificial intelligence for medicine and healthcare. Nat. Biomed. Eng..

[70] Mennella C., Maniscalco U., De Pietro G., Esposito M. (2024). Ethical and regulatory challenges of AI technologies in healthcare: A narrative review. Heliyon.

[71] Messina P., Pino P., Parra D., Soto A., Besa C., Uribe S., Andía M., Tejos C., Prieto C., Capurro D. (2022). A survey on deep learning and explainability for automatic report generation from medical images. ACM Comput. Surv..

[72] Paproki A., Salvado O., Fookes C. (2024). Synthetic data for deep learning in computer vision & medical imaging: A means to reduce data bias. ACM Comput. Surv..

[73] Hossain M.I., Zamzmi G., Mouton P.R., Salekin M.S., Sun Y., Goldgof D. (2025). Explainable AI for medical data: Current methods, limitations, and future directions. ACM Comput. Surv..

[74] Amann J., Bürger V.K., Livne M., Bui C.K., Madai V.I. (2025). The fundamentals of AI ethics in medical imaging. Trustworthy AI in Medical Imaging.

[75] Jordan M.I., Mitchell T.M. (2015). Machine learning: Trends, perspectives, and prospects. Science.

[76] Goisauf M., Cano Abadía M. (2022). Ethics of AI in radiology: A review of ethical and societal implications. Front. Big Data.

[77] Herington J., McCradden M.D., Creel K., Boellaard R., Jones E.C., Jha A.K., Rahmim A., Scott P.J., Sunderland J.J., Wahl R.L. (2023). Ethical considerations for artificial intelligence in medical imaging: Deployment and governance. J. Nucl. Med..

[78] Jiang L., Wu Z., Xu X., Zhan Y., Jin X., Wang L., Qiu Y. (2021). Opportunities and challenges of artificial intelligence in the medical field: Current application, emerging problems, and problem-solving strategies. J. Int. Med. Res..

[79] Theriault-Lauzier P., Cobin D., Tastet O., Langlais E.L., Taji B., Kang G., Chong A.Y., So D., Tang A., Gichoya J.W. (2024). A responsible framework for applying artificial intelligence on medical images and signals at the point-of-care: The PACS-AI platform. Can. J. Cardiol..

[80] Kulkov I. (2023). Next-generation business models for artificial intelligence start-ups in the healthcare industry. Int. J. Entrep. Behav. Res..

[81] Chikhaoui E., Alajmi A., Larabi-Marie-Sainte S. (2022). Artificial intelligence applications in healthcare sector: Ethical and legal challenges. Emerg. Sci. J..

[82] Saraswat D., Bhattacharya P., Verma A., Prasad V.K., Tanwar S., Sharma G., Bokoro P.N., Sharma R. (2022). Explainable AI for healthcare 5.0: Opportunities and challenges. IEEE Access.

[83] Aminizadeh S., Heidari A., Dehghan M., Toumaj S., Rezaei M., Navimipour N.J., Stroppa F., Unal M. (2024). Opportunities and challenges of artificial intelligence and distributed systems to improve the quality of healthcare service. Artif. Intell. Med..

[84] Tulgar Y.K., Tulgar S., Köse S.G., Köse H.C., Nasırlıer G.Ç., Doğan M., Thomas D.T. (2023). Anesthesiologists’ perspective on the use of artificial intelligence in ultrasound-guided regional anaesthesia in terms of medical ethics and medical education: A survey study. Eurasian J. Med..

[85] Karimian G., Petelos E., Evers S.M. (2022). The ethical issues of the application of artificial intelligence in healthcare: A systematic scoping review. AI Ethics.

[86] Nasir S., Khan R.A., Bai S. (2024). Ethical framework for harnessing the power of AI in healthcare and beyond. IEEE Access.

[87] Zahlan A., Ranjan R.P., Hayes D. (2023). Artificial intelligence innovation in healthcare: Literature review, exploratory analysis, and future research. Technol. Soc..

[88] Zangana H.M., Sallow Z.B., Salih B.A. (2025). The Impact of Artificial Intelligence on Healthcare: A Systematic Review of Innovations, Challenges, and Ethical Considerations. J. Comput. Digit. Bus..

[89] Almeida-Galárraga D., Tirado-Espín A. (2025). Acceptability of AI in Medical Diagnostics: A Discourse Analysis. Commun. Appl. Technol. Proc. ICOMTA 2024.

[90] Nowrozy R., Ahmed K., Wang H. (2025). GPT, ontology, and CAABAC: A tripartite personalized access control model anchored by compliance, context and attribute. PLoS ONE.

[91] Nowrozy R., Ahmed K., Wang H., Mcintosh T. (2023). Towards a universal privacy model for electronic health record systems: An ontology and machine learning approach. Informatics.

[92] Nowrozy R. (2024). A Security and Privacy Compliant Data Sharing Solution For Healthcare Data Ecosystems. Ph.D. Thesis.

[93] Shrotriya V., Jain M.A., Shrivastava P., Sharma P. (2019). Exploring IoT solutions for connecting and synchronizing various maternal health monitoring devices. Interdisciplinary Work of Science and Technology in Maternal and Child Care.

[94] Ntjamba F.C., Ashipala D.O. (2025). Impact on and Ethical Considerations of Artificial Intelligence on Human Healthcare. AI Technologies and Advancements for Psychological Well-Being and Healthcare.

[95] Pantelopoulos A., Bourbakis N.G. (2009). A survey on wearable sensor-based systems for health monitoring and prognosis. IEEE Trans. Syst. Man Cybern. Part C (Appl. Rev.).

[96] AlAmir M., AlGhamdi M. (2022). The Role of generative adversarial network in medical image analysis: An in-depth survey. ACM Comput. Surv..

[97] Stogiannos N., Georgiadou E., Rarri N., Malamateniou C. (2025). Ethical AI: A qualitative study exploring ethical challenges and solutions on the use of AI in medical imaging. Eur. J. Radiol. Artif. Intell..

[98] Wang J., Wang K., Yu Y., Lu Y., Xiao W., Sun Z., Liu F., Zou Z., Gao Y., Yang L. (2025). Self-improving generative foundation model for synthetic medical image generation and clinical applications. Nat. Med..

[99] Koçak B., Ponsiglione A., Stanzione A., Bluethgen C., Santinha J., Ugga L., Huisman M., Klontzas M.E., Cannella R., Cuocolo R. (2025). Bias in artificial intelligence for medical imaging: Fundamentals, detection, avoidance, mitigation, challenges, ethics, and prospects. Diagn. Interv. Radiol..

[100] Sand M., Durán J.M., Jongsma K.R. (2022). Responsibility beyond design: Physicians’ requirements for ethical medical AI. Bioethics.

[101] Nowrozy R., Ahmed K. (2024). A Systematic Survey on AI Governance in Healthcare. ACM Comput. Surv..

[102] Megerian J.T., Dey S., Melmed R.D., Coury D.L., Lerner M., Nicholls C.J., Sohl K., Rouhbakhsh R., Narasimhan A., Romain J. (2022). Evaluation of an artificial intelligence-based medical device for diagnosis of autism spectrum disorder. npj Digit. Med..

[103] Reddy S., Allan S., Coghlan S., Cooper P. (2020). A governance model for the application of AI in health care. J. Am. Med. Inform. Assoc..

[104] Abrámoff M.D., Roehrenbeck C., Trujillo S., Goldstein J., Graves A.S., Repka M.X., Silva E.Z. (2022). A reimbursement framework for artificial intelligence in healthcare. npj Digit. Med..

[105] Amjad A., Kordel P., Fernandes G. (2023). A review on innovation in healthcare sector (telehealth) through artificial intelligence. Sustainability.

[106] El-Sherif D.M., Abouzid M., Elzarif M.T., Ahmed A.A., Albakri A., Alshehri M.M. (2022). Telehealth and artificial intelligence insights into healthcare during the COVID-19 pandemic. Healthcare.

[107] Kotter E., D’Antonoli T.A., Cuocolo R., Hierath M., Huisman M., Klontzas M.E., Martí-Bonmatí L., May M.S., Neri E., Nikolaou K. (2025). Guiding AI in radiology: ESR’s recommendations for effective implementation of the European AI Act. Insights Into Imaging.

[108] Corfmat M., Martineau J.T., Régis C. (2025). High-reward, high-risk technologies? An ethical and legal account of AI development in healthcare. BMC Med. Ethics.

[109] Hickman S.E., Baxter G.C., Gilbert F.J. (2021). Adoption of artificial intelligence in breast imaging: Evaluation, ethical constraints and limitations. Br. J. Cancer.

[110] Dave T., Athaluri S.A., Singh S. (2023). ChatGPT in medicine: An overview of its applications, advantages, limitations, future prospects, and ethical considerations. Front. Artif. Intell..

[111] Kothinti R.R. (2024). Artificial intelligence in healthcare: Revolutionizing precision medicine, predictive analytics, and ethical considerations in autonomous diagnostics. World J. Adv. Res. Rev..

[112] Maleki Varnosfaderani S., Forouzanfar M. (2024). The role of AI in hospitals and clinics: Transforming healthcare in the 21st century. Bioengineering.

[113] Mörch C., Atsu S., Cai W., Li X., Madathil S., Liu X., Mai V., Tamimi F., Dilhac M., Ducret M. (2021). Artificial intelligence and ethics in dentistry: A scoping review. J. Dent. Res..

[114] Al-kfairy M., Mustafa D., Kshetri N., Insiew M., Alfandi O. (2024). Ethical challenges and solutions of generative AI: An interdisciplinary perspective. Informatics.

[115] Čartolovni A., Tomičić A., Mosler E.L. (2022). Ethical, legal, and social considerations of AI-based medical decision-support tools: A scoping review. Int. J. Med. Inform..

[116] Dhar T., Dey N., Borra S., Sherratt R.S. (2023). Challenges of deep learning in medical image analysis—Improving explainability and trust. IEEE Trans. Technol. Soc..

[117] Durán J.M., Jongsma K.R. (2021). Who is afraid of black box algorithms? On the epistemological and ethical basis of trust in medical AI. J. Med. Ethics.

[118] Ullah S., Li J., Chen J., Ali I., Khan S., Ahad A., Ullah F., Leung V.C. (2024). A Survey on Emerging Trends and Applications of 5G and 6G to Healthcare Environments. ACM Comput. Surv..

[119] Hertel R., Benlamri R. (2023). Deep learning techniques for COVID-19 diagnosis and prognosis based on radiological imaging. ACM Comput. Surv..

[120] Wood A., Najarian K., Kahrobaei D. (2020). Homomorphic encryption for machine learning in medicine and bioinformatics. ACM Comput. Surv..

[121] Paladugu P.S., Ong J., Nelson N., Kamran S.A., Waisberg E., Zaman N., Kumar R., Dias R.D., Lee A.G., Tavakkoli A. (2023). Generative adversarial networks in medicine: Important considerations for this emerging innovation in artificial intelligence. Ann. Biomed. Eng..

[122] Showrov A.A., Aziz M.T., Nabil H.R., Jim J.R., Kabir M.M., Mridha M., Asai N., Shin J. (2024). Generative adversarial networks (GANs) in medical imaging: Advancements, applications and challenges. IEEE Access.

[123] Shafik W. (2025). Generative Adversarial Networks: Security, Privacy, and Ethical Considerations. Generative Artificial Intelligence (AI) Approaches for Industrial Applications.

[124] Ranschaert E.R., Morozov S., Algra P.R. (2019). Artificial Intelligence in Medical Imaging: Opportunities, Applications and Risks.

[125] Koohi-Moghadam M., Bae K.T. (2023). Generative AI in medical imaging: Applications, challenges, and ethics. J. Med. Syst..

[126] Nazar M., Alam M.M., Yafi E., Su’ud M.M. (2021). A systematic review of human–computer interaction and explainable artificial intelligence in healthcare with artificial intelligence techniques. IEEE Access.

[127] Giordano C., Brennan M., Mohamed B., Rashidi P., Modave F., Tighe P. (2021). Accessing artificial intelligence for clinical decision-making. Front. Digit. Health.

[128] Bartoletti I. (2019). AI in healthcare: Ethical and privacy challenges. Proceedings of the Artificial Intelligence in Medicine: 17th Conference on Artificial Intelligence in Medicine, AIME 2019.

[129] Blease C., Kaptchuk T.J., Bernstein M.H., Mandl K.D., Halamka J.D., DesRoches C.M. (2019). Artificial intelligence and the future of primary care: Exploratory qualitative study of UK general practitioners’ views. J. Med. Internet Res..

[130] Murphy K., Di Ruggiero E., Upshur R., Willison D.J., Malhotra N., Cai J.C., Malhotra N., Lui V., Gibson J. (2021). Artificial intelligence for good health: A scoping review of the ethics literature. BMC Med. Ethics.

[131] Lee E.E., Torous J., De Choudhury M., Depp C.A., Graham S.A., Kim H.C., Paulus M.P., Krystal J.H., Jeste D.V. (2021). Artificial intelligence for mental health care: Clinical applications, barriers, facilitators, and artificial wisdom. Biol. Psychiatry Cogn. Neurosci. Neuroimaging.

[132] Huang S., Yang J., Fong S., Zhao Q. (2020). Artificial intelligence in cancer diagnosis and prognosis: Opportunities and challenges. Cancer Lett..

[133] Mishra R., Satpathy R., Pati B. (2024). Interpretable AI in Medical Imaging: Enhancing Diagnostic Accuracy through Human–Computer Interaction. J. Artif. Intell. Syst..

[134] Patrício C., Neves J.C., Teixeira L.F. (2023). Explainable deep learning methods in medical image classification: A survey. ACM Comput. Surv..

[135] Jeyaraman M., Balaji S., Jeyaraman N., Yadav S. (2023). Unraveling the ethical enigma: Artificial intelligence in healthcare. Cureus.

[136] Zhu W., Huang L., Zhou X., Li X., Shi G., Ying J., Wang C. (2025). Could AI ethical anxiety, perceived ethical risks and ethical awareness about AI influence university students’ use of generative AI products? An ethical perspective. Int. J. Hum.-Comput. Interact..

[137] Müller H., Mayrhofer M.T., Van Veen E.B., Holzinger A. (2021). The Ten Commandments of Ethical Medical AI. Computer.

[138] Borenstein J., Howard A. (2021). Emerging challenges in AI and the need for AI ethics education. AI Ethics.

[139] Odle T. (2020). The AI era: The role of medical imaging and radiation therapy professionals. Radiol. Technol..

[140] Li J., Zhu G., Hua C., Feng M., Bennamoun B., Li P., Lu X., Song J., Shen P., Xu X. (2023). A systematic collection of medical image datasets for deep learning. ACM Comput. Surv..

[141] Dong J., Chen J., Xie X., Lai J., Chen H. (2024). Survey on Adversarial Attack and Defense for Medical Image Analysis: Methods and Challenges. ACM Comput. Surv..

[142] Thieme A., Rajamohan A., Cooper B., Groombridge H., Simister R., Wong B., Woznitza N., Pinnock M.A., Wetscherek M.T., Morrison C. (2025). Challenges for Responsible AI Design and Workflow Integration in Healthcare: A Case Study of Automatic Feeding Tube Qualification in Radiology. ACM Trans. Comput.-Hum. Interact..

[143] Chauhan C., Gullapalli R.R. (2025). Ethics of AI in pathology: Current paradigms and emerging issues. Artificial Intelligence in Pathology.

[144] D’Antonoli T.A. (2020). Ethical considerations for artificial intelligence: An overview of the current radiology landscape. Diagn. Interv. Radiol..

[145] Naik N., Hameed B., Shetty D.K., Swain D., Shah M., Paul R., Aggarwal K., Ibrahim S., Patil V., Smriti K. (2022). Legal and ethical consideration in artificial intelligence in healthcare: Who takes responsibility?. Front. Surg..

[146] Pesapane F., Hauglid M.K., Fumagalli M., Petersson L., Parkar A.P., Cassano E., Horgan D. (2025). The translation of in-house imaging AI research into a medical device ensuring ethical and regulatory integrity. Eur. J. Radiol..

[147] Wang P., Gao Q., Wu X., You T., Du H., Wang X., Zeng S., Lv Q., Ding X., Wang L. (2024). Ethics and Safety in Medical Imaging and Artificial Intelligence. Artificial Intelligence in Medical Imaging in China.

[148] Currie G., Hawk K.E., Rohren E.M. (2020). Ethical principles for the application of artificial intelligence (AI) in nuclear medicine. Eur. J. Nucl. Med. Mol. Imaging.

[149] Arkoh S., Akudjedu T.N., Amedu C., Antwi W.K., Elshami W., Ohene-Botwe B. (2025). Current Radiology workforce perspective on the integration of artificial intelligence in clinical practice: A systematic review. J. Med. Imaging Radiat. Sci..

[150] Priyadarshi R., Ranjan R., Vishwakarma A.K., Yang T., Rathore R.S. (2024). Exploring the Frontiers of Unsupervised Learning Techniques for Diagnosis of Cardiovascular Disorder: A Systematic Review. IEEE Access.

[151] Ganesan S., Somasiri N. (2024). Navigating the Integration of Machine Learning in Healthcare: Challenges, Strategies, and Ethical Considerations. J. Comput. Cogn. Eng..

[152] Nanjundan P., Indu P., Thomas L. (2025). Navigating the ethical landscape of artificial intelligence: Challenges, frameworks, and responsible deployment. Artificial Intelligence Technologies for Engineering Applications.

[153] Ji S., Li X., Sun W., Dong H., Taalas A., Zhang Y., Wu H., Pitkänen E., Marttinen P. (2024). A unified review of deep learning for automated medical coding. ACM Comput. Surv..

[154] Mohsin Khan M., Shah N., Shaikh N., Thabet A., alrabayah T., Belkhair S. (2025). Towards secure and trusted AI in healthcare: A systematic review of emerging innovations and ethical challenges. Int. J. Med. Inform..

[155] Saw S.N., Ng K.H. (2022). Current challenges of implementing artificial intelligence in medical imaging. Phys. Medica.

[156] Aldhafeeri F.M. (2025). Governing Artificial Intelligence in Radiology: A Systematic Review of Ethical, Legal, and Regulatory Frameworks. Diagnostics.

