# Supplementary material for: Ethical and Governance Challenges of AI in Medical Imaging and Diagnostics: A Systematic Survey and Policy Framework Recommendations
Source: Healthcare (Basel). 2026 Jul 2;14(13):1975. doi: 10.3390/healthcare14131975 (PMC13361511; doi:10.3390/healthcare14131975)
Supplement: Supplementary file 1 [file healthcare-14-01975-s001.zip › Supplementary File S3.pdf]

Supplementary File S3

Included Studies and Thematic Classification Matrix (Excerpt)

Ref. No.	Citation (First Author, Year)	Evidence Type	Primary Theme	Secondary Theme
**1**	Bi et al. (2019) [1]	Clinical Review	Clinical Integration and Trust	Cancer Imaging Applications
**2**	Habli et al. (2020) [2]	Conceptual/Ethical Analysis	Accountability and Liability	Safety and Governance
**3**	Guo et al. (2020) [3]	Bibliometric Analysis	Regulation and Governance	Research Trends
**4**	Jimma (2023) [4]	Bibliometric Analysis	Regulation and Governance	Research Trends
**5**	Chen and Decary (2020) [5]	Professional Guidance/Report	Clinical Integration and Trust	Healthcare Leadership
**6**	Kumar et al. (2023) [6]	Conceptual/Ethical Analysis	Ethics and Trust	Explainability and Accountability
**7**	Holmes et al. (2004) [7]	Conceptual Review	Clinical Integration and Trust	AI Adoption
**8**	Liu et al. (2021) [8]	Narrative Review	Clinical Integration and Trust	AI Applications
**9**	Beam et al. (2023) [9]	Commentary/Perspective	Regulation and Governance	Clinical Adoption
**10**	Farhud and Zokaei (2021) [10]	Conceptual/Ethical Analysis	Ethics and Trust	Accountability and Privacy
**11**	Bekbolatova et al. (2024) [11]	Conceptual Review	Ethics and Trust	Public Perceptions

**12**	Weiner et al. (2025) [12]	Conceptual/Ethical Analysis	Clinical Governance	Ethical Integration
**13**	Najjar (2023) [13]	Narrative Review	Clinical Integration and Trust	Medical Imaging Applications
**14**	Ahmad et al. (2021) [14]	Narrative Review	Clinical Integration and Trust	Pathology Applications
**15**	Barragán-Montero et al. (2021) [15]	Technology Review	Clinical Integration and Trust	Medical Imaging Applications
**16**	Huynh et al. (2020) [16]	Clinical Review	Clinical Integration and Trust	Radiation Oncology
**17**	Thompson et al. (2018) [17]	Clinical Review	Clinical Integration and Trust	Radiation Oncology
**18**	Ahmed et al. (2020) [18]	Technology Review	Clinical Integration and Trust	Precision Medicine
**19**	Vaisman et al. (2020) [19]	Conceptual/Ethical Analysis	Ethics and Trust	Diagnostic Imaging Ethics
**20**	Petersson et al. (2022) [20]	Empirical Study (Qualitative Interviews)	Clinical Governance	Implementation Barriers
**21**	Iqbal et al. (2021) [21]	Clinical Review	Clinical Integration and Trust	Cancer Diagnosis
**22**	Beil et al. (2019) [22]	Conceptual/Ethical Analysis	Ethics and Trust	Prognostication and Decision-Making
**23**	McCradden et al. (2020) [23]	Conceptual/Ethical Analysis	Bias and Fairness	Algorithmic Fairness
**24**	Chen et al. (2021) [24]	Narrative Review	Ethics and Trust	Responsible AI Development
**25**	Rasheed et al. (2022) [25]	Systematic Survey	Explainability and Transparency	Trustworthy AI

**26**	Albahri et al. (2023) [26]	Systematic Review	Explainability and Transparency	Bias Assessment and Trustworthiness
**27**	Kaissis et al. (2020) [27]	Technology Review	Privacy and Data Governance	Federated Learning
**28**	Jobin et al. (2019) [28]	Comparative Policy Analysis	Regulation and Governance	AI Ethics Guidelines
**29**	Shaheen (2021) [29]	Narrative Review	Clinical Integration and Trust	Healthcare Applications
**30**	Sun et al. (2025) [30]	Narrative Review	Explainability and Transparency	Explainable AI
**31**	Char et al. (2020) [31]	Conceptual/Ethical Analysis	Ethics and Trust	Machine Learning Governance
**32**	Zhang and Zhang (2023) [32]	Conceptual/Ethical Analysis	Regulation and Governance	Trustworthy Medical AI
**33**	Geis et al. (2019) [33]	Professional Guidance/Consensus Statement	Ethics and Trust	Radiology Governance
**34**	Amann et al. (2020) [34]	Narrative Review	Explainability and Transparency	Interpretability in Healthcare AI
**35**	Goirand et al. (2021) [35]	Scoping Review	Ethics and Trust	AI Ethics Implementation
**36**	Recht et al. (2020) [36]	Professional Guidance/Expert Review	Clinical Governance	Radiology Implementation
**37**	Nichols et al. (2019) [37]	Narrative Review	Clinical Integration and Trust	Medical Imaging Applications

**38**	Sun and Medaglia (2019) [38]	Empirical Review	Clinical Governance	Healthcare Implementation Challenges
**39**	Grunhut et al. (2022) [39]	Narrative Review	Clinical Integration and Trust	AI Education and Workforce Development
**40**	Reyes et al. (2020) [40]	Narrative Review	Explainability and Transparency	Interpretability in Radiology
**41**	Chew and Achananuparp (2022) [41]	Scoping Review	Clinical Integration and Trust	Adoption and Stakeholder Perceptions
**42**	Laï et al. (2020) [42]	Empirical Study (Qualitative Survey)	Clinical Integration and Trust	Public and Professional Perceptions
**43**	Olczak et al. (2021) [43]	Professional Guidance/Checklist Development	Regulation and Governance	Reporting Standards and Transparency
**44**	Khalid et al. (2023) [44]	Narrative Review	Privacy and Data Governance	Privacy-Preserving AI
**45**	Larson et al. (2021) [45]	Professional Guidance/Policy Analysis	Regulation and Governance	Regulatory Frameworks for Medical Imaging AI
**46**	Bouderhem (2024) [46]	Conceptual/Ethical Analysis	Regulation and Governance	Ethics and Governance Frameworks
**47**	Arnold (2021) [47]	Conceptual/Ethical Analysis	Ethics and Trust	Ethical Critique of AI in Medicine
**48**	Morley et al. (2020) [48]	Mapping Review	Ethics and Trust	AI Ethics Landscape
**49**	Li et al. (2022) [49]	Systematic Review	Ethics and Trust	Ethical Design Strategies

**50**	Siala and Wang (2022) [50]	Systematic Review	Regulation and Governance	Responsible AI Governance
**51**	Ahuja (2019) [51]	Narrative Review	Clinical Integration and Trust	Future Role of Physicians
**52**	Doraiswamy et al. (2020) [52]	Empirical Study (Global Physician Survey)	Clinical Integration and Trust	Physician Perceptions of AI
**53**	Krittanawong (2018) [53]	Commentary/Perspective	Clinical Integration and Trust	Future Role of Physicians
**54**	Yan et al. (2019) [54]	Narrative Review	Clinical Integration and Trust	Cardiovascular Applications
**55**	Grunhut et al. (2021) [55]	Integrative Review	Clinical Integration and Trust	AI Education and Workforce Preparedness
**56**	Zuhair et al. (2024) [56]	Narrative Review	Clinical Integration and Trust	Global Health and Developing Nations
**57**	Gala et al. (2024) [57]	Narrative Review	Clinical Integration and Trust	Cardiology and Patient Outcomes
**58**	Blease et al. (2024) [58]	Empirical Study (Mixed Methods Survey)	Clinical Integration and Trust	Generative AI and Clinician Perceptions
**59**	Grzybowski et al. (2024) [59]	Narrative Review	Clinical Governance	Challenges of AI Implementation
**60**	Johnson et al. (2023) [60]	Narrative Review	Clinical Governance	Benefits and Risks of Clinical AI
**61**	Aung et al. (2021) [61]	Narrative Review	Clinical Integration and Trust	Opportunities and Challenges

**62**	Wubineh et al. (2024) [62]	Systematic Literature Review	Clinical Governance	Implementation Opportunities and Barriers
**63**	Olawade et al. (2024) [63]	Narrative Review	Clinical Governance	Healthcare Delivery Prospects and Pitfalls
**64**	Bhagat and Kanyal (2024) [64]	Comprehensive Review	Clinical Governance	Hospital Management and Digital Transformation
**65**	Bellini et al. (2024) [65]	Narrative Review	Clinical Governance	Operating Room Management
**66**	Khang (2024) [66]	Book/Technical Reference	Privacy and Data Governance	Smart Healthcare Systems and IoT
**67**	Chen et al. (2024) [67]	Bibliometric Analysis	Clinical Governance	Multimodal Data and Smart Healthcare
**68**	Pradyumna et al. (2024) [68]	Technical Review	Privacy and Data Governance	Security, Interoperability, and IoMT
**69**	Chen et al. (2023) [69]	Narrative Review	Bias and Fairness	Algorithmic Fairness in Healthcare AI
**70**	Mennella et al. (2024) [70]	Narrative Review	Regulation and Governance	Ethical and Regulatory Challenges
**71**	Messina et al. (2022) [71]	Survey Review	Explainability and Transparency	Automated Report Generation
**72**	Paproki et al. (2024) [72]	Survey Review	Bias and Fairness	Synthetic Data and Bias Mitigation

**73**	Hossain et al. (2025) [73]	Survey Review	Explainability and Transparency	Explainable AI Methods
**74**	Amann et al. (2025) [74]	Book Chapter / Conceptual Analysis	Ethics and Trust	AI Ethics in Medical Imaging
**75**	Jordan and Mitchell (2015) [75]	Foundational Perspective Review	Clinical Integration and Trust	Machine Learning Foundations
**76**	Goisauf and Cano Abadía (2022) [76]	Narrative Review	Ethics and Trust	Ethical and Societal Implications in Radiology
**77**	Herington et al. (2023) [77]	Expert Guidance / Policy Analysis	Regulation and Governance	Deployment and Governance of Medical Imaging AI
**78**	Jiang et al. (2021) [78]	Narrative Review	Clinical Governance	Opportunities, Challenges, and Implementation Strategies
**79**	Theriault-Lauzier et al. (2024) [79]	Framework / Implementation Study	Regulation and Governance	Responsible AI Frameworks
**80**	Kulkov (2023) [80]	Empirical Policy Analysis	Regulation and Governance	Innovation and Healthcare Business Models
**81**	Chikhaoui et al. (2022) [81]	Narrative Review	Regulation and Governance	Ethical and Legal Challenges
**82**	Saraswat et al. (2022) [82]	Technical Review	Explainability and Transparency	Explainable AI for Healthcare 5.0
**83**	Aminizadeh et al. (2024) [83]	Narrative Review	Clinical Governance	AI Implementation

				and Distributed Systems
**84**	Tulgar et al. (2023) [84]	Empirical Study (Survey)	Clinical Integration and Trust	Clinician Perspectives and Medical Ethics
**85**	Karimian et al. (2022) [85]	Systematic Scoping Review	Ethics and Trust	Ethical Issues in Healthcare AI
**86**	Nasir et al. (2024) [86]	Framework / Conceptual Analysis	Regulation and Governance	Ethical Governance Frameworks
**87**	Zahlan et al. (2023) [87]	Literature Review	Clinical Governance	AI Innovation and Future Research
**88**	Zangana et al. (2025) [88]	Systematic Review	Ethics and Trust	Innovations, Challenges, and Ethical Considerations
**89**	Almeida-Galárraga and Tirado-Espín (2025) [89]	Discourse Analysis	Clinical Integration and Trust	Acceptability of AI Diagnostics
**90**	Nowrozy et al. (2025) [90]	Technical Framework Study	Privacy and Data Governance	Access Control and Compliance
**91**	Nowrozy et al. (2023) [91]	Technical Framework Study	Privacy and Data Governance	Electronic Health Record Privacy
**92**	Nowrozy (2024) [92]	Doctoral Thesis	Privacy and Data Governance	Secure Healthcare Data Sharing
**93**	Shrotriya et al. (2019) [93]	Technical Application Study	Clinical Governance	IoT-Based Health Monitoring
**94**	Ntjamba and Ashipala (2025) [94]	Book Chapter / Conceptual Analysis	Ethics and Trust	Ethical Implications of AI in Healthcare

**95**	Pantelopoulos and Bourbakis (2009) [95]	Survey Review	Clinical Integration and Trust	Wearable Health Monitoring Systems
**96**	AlAmir and AlGhamdi (2022) [96]	Survey Review	Clinical Integration and Trust	Generative AI and Medical Imaging
**97**	Stogiannos et al. (2025) [97]	Empirical Study (Qualitative Study)	Ethics and Trust	Ethical Challenges in Medical Imaging
**98**	Wang et al. (2025) [98]	Empirical Study / Technical Development	Clinical Integration and Trust	Synthetic Medical Image Generation
**99**	Koçak et al. (2025) [99]	Narrative Review	Bias and Fairness	Bias Detection and Mitigation in Medical Imaging
**100**	Sand et al. (2022) [100]	Conceptual/Ethical Analysis	Human Oversight and Accountability	Physician Responsibility and Ethical AI
**101**	Nowrozy and Ahmed (2024) [101]	Systematic Survey	Regulation and Governance	AI Governance in Healthcare
**102**	Megerian et al. (2022) [102]	Clinical Validation Study	Clinical Governance	AI Medical Device Evaluation
**103**	Reddy et al. (2020) [103]	Governance Framework Paper	Regulation and Governance	AI Governance Models
**104**	Abràmoff et al. (2022) [104]	Policy Analysis	Regulation and Governance	Reimbursement and Health System Adoption
**105**	Amjad et al. (2023) [105]	Review Article	Clinical Integration and Trust	Telehealth Innovation

**106**	El-Sherif et al. (2022) [106]	Review Article	Clinical Integration and Trust	Telehealth and AI During COVID-19
**107**	Kotter et al. (2025) [107]	Policy and Regulatory Guidance	Regulation and Governance	European AI Act Implementation
**108**	Corfmat et al. (2025) [108]	Ethical and Legal Analysis	Regulation and Governance	Ethical and Legal Risks of AI Development
**109**	Hickman et al. (2021) [109]	Review Article	Clinical Governance	AI Adoption in Medical Imaging
**110**	Dave et al. (2023) [110]	Narrative Review	Ethics and Trust	Generative AI in Medicine
**111**	Kothinti (2024) [111]	Review Article	Ethics and Trust	Precision Medicine and Ethical Considerations
**112**	Maleki Varnosfaderani and Forouzanfar (2024) [112]	Review Article	Clinical Integration and Trust	AI Transformation of Hospitals and Clinics
**113**	Mörch et al. (2021) [113]	Scoping Review	Ethics and Trust	AI Ethics in Clinical Practice
**114**	Al-kfairy et al. (2024) [114]	Conceptual Analysis	Ethics and Trust	Ethical Challenges of Generative AI
**115**	Čartolovni et al. (2022) [115]	Scoping Review	Human Oversight and Accountability	AI-Based Clinical Decision Support
**116**	Dhar et al. (2023) [116]	Technical Review	Explainability and Transparency	Trustworthy Medical AI
**117**	Durán and Jongsma (2021) [117]	Conceptual Analysis	Explainability and Transparency	Trust and Black-Box Algorithms

**118**	Ullah et al. (2024) [118]	Survey Review	Clinical Integration and Trust	Digital Health Infrastructure
**119**	Hertel and Benlamri (2023) [119]	Survey Review	Clinical Integration and Trust	Deep Learning in Medical Imaging
**120**	Wood et al. (2020) [120]	Technical Survey	Privacy and Data Governance	Homomorphic Encryption
**121**	Paladugu et al. (2023) [121]	Review Article	Clinical Governance	Generative Adversarial Networks in Medicine
**122**	Showrov et al. (2024) [122]	Technical Review	Privacy and Data Governance	Synthetic Data and GAN Applications
**123**	Shafik (2025) [123]	Conceptual Analysis	Privacy and Data Governance	Security, Privacy and Ethical Risks
**124**	Ranschaert et al. (2019) [124]	Edited Book / Reference Text	Clinical Governance	Opportunities, Risks and AI Implementation
**125**	Koohi-Moghadam and Bae (2023) [125]	Review Article	Ethics and Trust	Generative AI in Medical Imaging
**126**	Nazar et al. (2021) [126]	Systematic Review	Explainability and Transparency	Human–Computer Interaction and XAI
**127**	Giordano et al. (2021) [127]	Review Article	Human Oversight and Accountability	Clinical Decision-Making Support
**128**	Bartoletti (2019) [128]	Conference Paper	Privacy and Data Governance	Ethical and Privacy Challenges
**129**	Blease et al. (2019) [129]	Qualitative Study	Clinical Integration and Trust	Clinician Perspectives and Adoption

**130**	Murphy et al. (2021) [130]	Scoping Review	Ethics and Trust	AI Ethics in Healthcare
**131**	Lee et al. (2021) [131]	Review Article	Clinical Integration and Trust	Barriers and Facilitators to Adoption
**132**	Huang et al. (2020) [132]	Review Article	Clinical Governance	AI in Cancer Diagnosis and Prognosis
**133**	Mishra et al. (2024) [133]	Conceptual / Technical Review	Explainability and Transparency	Human–Computer Interaction
**134**	Patrício et al. (2023) [134]	Survey Review	Explainability and Transparency	Explainable Deep Learning
**135**	Jeyaraman et al. (2023) [135]	Review Article	Ethics and Trust	Ethical Challenges in Healthcare AI
**136**	Zhu et al. (2025) [136]	Empirical Study	Ethics and Trust	Ethical Risk Perception and AI Use
**137**	Müller et al. (2021) [137]	Conceptual Framework	Ethics and Trust	Ethical Principles for Medical AI
**138**	Borenstein and Howard (2021) [138]	Commentary / Perspective	Ethics and Trust	AI Ethics Education
**139**	Odle (2020) [139]	Professional Perspective	Clinical Integration and Trust	Workforce Implications
**140**	Li et al. (2023) [140]	Systematic Survey	Privacy and Data Governance	Medical Imaging Datasets and Data Quality
**141**	Dong et al. (2024) [141]	Survey Review	Privacy and Data Governance	Adversarial Attacks and AI Security

**142**	Thieme et al. (2025) [142]	Case Study	Clinical Integration and Trust	Responsible AI Implementation
**143**	Chauhan and Gullapalli (2025) [143]	Book Chapter	Ethics and Trust	AI Ethics in Pathology
**144**	D'Antonoli (2020) [144]	Review Article	Ethics and Trust	Ethical Issues in Radiology AI
**145**	Naik et al. (2022) [145]	Review Article	Human Oversight and Accountability	Legal Responsibility and Liability
**146**	Pesapane et al. (2025) [146]	Translational / Regulatory Analysis	Regulation and Governance	Medical Device Regulation
**147**	Wang et al. (2024) [147]	Book Chapter	Ethics and Trust	Ethics and Safety in Medical Imaging
**148**	Currie et al. (2020) [148]	Perspective Article	Ethics and Trust	Ethical Principles in Nuclear Medicine
**149**	Arkoh et al. (2025) [149]	Systematic Review	Clinical Integration and Trust	Workforce Perspectives
**150**	Priyadarshi et al. (2024) [150]	Systematic Review	Clinical Integration and Trust	AI Applications in Diagnostics
**151**	Ganesan et al. (2024) [151]	Review Article	Ethics and Trust	Implementation Challenges and Strategies
**152**	Nanjundan et al. (2025) [152]	Conceptual Framework	Regulation and Governance	Responsible AI Frameworks
**153**	Ji et al. (2024) [153]	Review Article	Clinical Integration and Trust	Healthcare Workflow Automation

**154**	Mohsin Khan et al. (2025) [154]	Systematic Review	Privacy and Data Governance	Secure and Trusted AI
**155**	Saw and Ng (2022) [155]	Review Article	Clinical Integration and Trust	Implementation Challenges in Medical Imaging
**156**	Aldhafeeri (2025) [156]	Systematic Review	Regulation and Governance	Ethical, Legal and Regulatory Frameworks
