# Supplementary material for: Ethical and Governance Challenges of AI in Medical Imaging and Diagnostics: A Systematic Survey and Policy Framework Recommendations
Source: Healthcare (Basel). 2026 Jul 2;14(13):1975. doi: 10.3390/healthcare14131975 (PMC13361511; doi:10.3390/healthcare14131975)
Supplement: Supplementary file 1 [file healthcare-14-01975-s001.zip › Supplementary File S1.pdf]

Supplementary File S1

PRISMA-ScR Reporting Checklist
Manuscript Title: Ethical and Governance Challenges of AI in Medical Imaging and Diagnostics: A Systematic Survey and Policy Framework Recommendations

Section	Item	PRISMA-ScR Reporting Element	Location in Manuscript
Title	1	Identification of review approach and reporting framework	Title, Abstract
Abstract	2	Structured summary including background, objectives, methods, results, and conclusions	Abstract
Introduction	3	Rationale for the review	Introduction
Introduction	4	Objectives and research questions	Section 2.1
Methods	5	Review design and reporting framework	Section 2.1
Methods	6	Eligibility criteria	Section 2.3
Methods	7	Information sources	Section 2.2
Methods	8	Search strategy	Sections 2.2 and 2.11, Supplementary File S2
Methods	9	Screening and selection process	Section 2.5
Methods	10	Data extraction and classification procedures	Sections 2.5-2.8
Methods	11	Data items and evidence categorisation	Sections 2.6 and 2.9
Methods	12	Critical appraisal approach	Section 2.9
Methods	13	Synthesis methodology	Section 2.8
Results	14	Selection of evidence sources	Section 2.7, Figure 4
Results	15	Characteristics of included sources	Section 3.1
Results	16	Evidence classification and thematic domains	Sections 3.1-3.6
Results	17	Presentation of findings	Sections 3.1-3.6
Results	18	Synthesis of evidence	Sections 3.1-3.6
Discussion	19	Summary of principal findings	Section 4.1

Discussion	20	Limitations	Section 4.3
Discussion	21	Conclusions and implications	Section 5
Supplementary Materials	22	Database-specific search strategies	Supplementary File S2
Supplementary Materials	23	Included source matrix and thematic classifications	Supplementary File S3

Note:

This review was conducted as a systematic survey informed by PRISMA-ScR reporting guidance. PRISMA-ScR was used to enhance reporting transparency, methodological traceability, and completeness because the review synthesised a heterogeneous body of evidence comprising empirical studies, technical papers, regulatory documents, policy reports, professional guidance materials, and conceptual analyses. The review was not conducted as a formal scoping review; rather, PRISMA-ScR reporting principles were adopted to support transparent reporting of a broad interdisciplinary evidence synthesis.
