# Supplementary material for: Ethical and Governance Challenges of AI in Medical Imaging and Diagnostics: A Systematic Survey and Policy Framework Recommendations
Source: Healthcare (Basel). 2026 Jul 2;14(13):1975. doi: 10.3390/healthcare14131975 (PMC13361511; doi:10.3390/healthcare14131975)
Supplement: Supplementary file 1 [file healthcare-14-01975-s001.zip › Supplementary File S2.pdf]

Supplementary File S2

Database-Specific Search Strategies and Search Dates

This supplementary file provides the database-specific search strategies used to guide the literature search process. Search terms were adapted where necessary to accommodate differences in database indexing systems, search interfaces, and controlled vocabularies while maintaining consistency with the review objectives and eligibility criteria.

Table S2. Database-specific search strategies and search dates used during the review process.

Database	Search Strategy Reference	Search Date
PubMed/MEDLINE	Search Strategy 1	April 2025
ACM Digital Library	Search Strategy 2	April 2025
IEEE Xplore	Search Strategy 3	April 2025
SpringerLink	Search Strategy 4	April 2025
ScienceDirect	Search Strategy 5	April 2025
Google Scholar	Search Strategy 6	April 2025

Search Strategy 1 – PubMed/MEDLINE

("artificial intelligence" OR "machine learning" OR "deep learning" OR "large language models" OR LLM OR CNN)

AND

("medical imaging" OR radiology OR diagnostics OR "diagnostic imaging")

AND

(ethics OR governance OR fairness OR explainability OR transparency OR accountability OR privacy)

AND

(healthcare OR clinical OR hospital)

Search Strategy 2 – ACM Digital Library

("artificial intelligence" OR "machine learning" OR "deep learning")

AND

("medical imaging" OR radiology OR diagnostics)

AND

(ethics OR governance OR explainability OR accountability)

Search Strategy 3 – IEEE Xplore

("artificial intelligence" OR "machine learning" OR "deep learning")

AND

("medical imaging" OR radiology OR diagnostics)

AND

(ethics OR governance OR fairness OR transparency OR accountability)

Search Strategy 4 – SpringerLink

("artificial intelligence")

AND

("medical imaging" OR diagnostics)

AND

(ethics OR governance OR accountability)

Search Strategy 5 – ScienceDirect

("artificial intelligence" OR "machine learning")

AND

("medical imaging" OR radiology)

AND

(ethics OR governance OR regulation)

Search Strategy 6 – Google Scholar

("artificial intelligence" OR "machine learning")
AND
("medical imaging" OR radiology)
AND
(ethics OR governance)
